# Modeling the dynamics of leptospirosis in India

**DOI:** 10.1038/s41598-023-46326-2

**Published:** 2023-11-13

**Authors:** Antima, Sandip Banerjee

**Affiliations:** Department of Mathematics, Indian Institute of Technology Roorkee, Roorkee, Uttarakhand 247667 India

**Keywords:** Bacterial infection, Applied mathematics

## Abstract

Leptospirosis, a formidable zoonotic threat spawned by *Leptospira*, plagues tropical and subtropical realms. This study delves deep into tropical Indian states, namely, Kerala, Gujarat, Karnataka, Maharashtra, and Tamil Nadu, unraveling the dynamics of leptospirosis through a comprehensive mathematical model that embraces temperature-driven growth rates of *Leptospira*. Sensitivity analysis and parameter estimation techniques fortified the model’s accuracy, unraveling the factors shaping leptospirosis transmission. Notably, the numerical results highlight the significant impact of rainfall, fishing, climate, mining, agriculture, and cattle farming on leptospirosis prevalence in the endemic states of India. Finally, our study urges resolute preventive action to control and combat leptospirosis in India. Strengthening surveillance, impactful awareness campaigns, targeted interventions, and improved hygiene practices among high-risk individuals are vital. Embracing these proactive strategies will alleviate the burden of leptospirosis and enhance public health in India and beyond.

## Introduction

Controlling and predicting zoonotic diseases requires integrating humans, animals, and the environment. More prominent inclusion of environmental and ecosystem components has been emphasized as a missing link in the OneHealth approach^1^ while improving investment in animal and human interfaces. Leptospirosis is a zoonotic disease that is globally widespread in countries with humid subtropical or tropical climates due to excessive rainfall and flooding. It also lacks adequate investigations linking animal and environmental factors to human infection and has the pandemic potential^2–4^. Leptospirosis is an acute bacterial infection induced by spirochetes with diverse pathogenic *Leptospira* species^5^. Spirochetes grow in different organs, most notably the liver, kidneys, and the central nervous system. They are eliminated by the immune system from the blood and the majority of tissues, but they can persist for days to months in freshwater, mud, soil, or hot and humid environments. It can be either pathogenic or saprophytic. The pathogen is transmitted through contact with contaminated water or urine of infected animals. Most mammalian species, cats, rodents, dogs, livestock, pigs, cattle, horses, rare bats, and wild animals are natural carriers of the pathogen *leptospires*, and living close to humans, presents a potentially high-risk reservoir^6,7^. *Leptospires* have a commensal relationship with hosts, causing inconsequential or no prominent harm^8^. However, they may persist in excreting bacteria into the atmosphere continuously or periodically for a few months to several years. Because of this, livestock farming and agricultural practices are important epidemiological factors that influence the spread of leptospirosis infection in humans, and it has a significant financial impact on the meat and dairy industries.

Humans accidentally become infected with leptospirosis by consuming contaminated food or water. Cuts or abrasions on the skin or mucous membranes can permit bacteria to enter the body (eyes, nose, mouth, and sinuses). *Leptospires* in the environment are killed by desiccation (dehydration). *Leptospires* have a greater probability of coming into contact with people if they can endure longer in their surroundings. The survival of *leptospires* in the environment and, consequently, the spread of leptospirosis are influenced by soil moisture, surface water, temperature, and humidity. In humans, symptoms are generally headache, jaundice, conjunctivitis, myalgia, bleeding tendency, and pulmonary manifestations such as breathlessness, hemoptysis, and cough. The anicteric form of the disease can cause renal failure and liver damage^6^.

According to the World Health Organization’s (WHO) leptospirosis Burden Epidemiology Reference Group, 1.03 million cases of leptospirosis are reported worldwide each year, with 58,900 deaths, with the incidence in the tropics being approximately ten times higher than in temperate regions. Many outbreaks have been reported worldwide following natural disasters in urban areas, including Guyana^9^, India, Italy, Indonesia, Malaysia, and Philippines^10^. Reports of endemic transmission of leptospirosis are also reported from different regions, including Thailand^11^, Italy, New Caledonia^12^, Vietnam, Netherlands, Brazil^13^, Lao, Mexico, Germany^14^, New Zealand, Argentina, Sri Lanka^15^, California^16^, and Nicaragua. Leptospirosis has been a worldwide disease for decades. However, people in many countries are unfamiliar with leptospirosis and have insufficient knowledge of its source of infection, transmission route, risk factors, and preventive measures.

India is a leptospirosis hotspot. Leptospirosis infection causes significant mortality and morbidity despite underestimation and under-diagnosis due to a lack of awareness of the disease and a deficiency of proper laboratory diagnostic facilities in most regions of the country^17–19^. Paddy farming, livestock farming, and working in underground sewers are India’s most common modes of exposure to leptospirosis. Because of a lack of public awareness, the disease’s concern has not been fully addressed, even though it has been recognized for decades. In the late 1980s, a puzzling febrile malady began to surface in seasonal outbreaks, presenting a particularly alarming symptom: severe haemoptysis in the majority of cases. This affliction demonstrated epidemic potential, accompanied by alarmingly high case fatality rates ranging from $10$ to $50\%$. It became known as Andaman Haemorrhagic Fever (AHF), a name coined to capture its enigmatic nature, as its underlying cause remained shrouded in mystery for a prolonged 5-year period^20^. In 1995, a breakthrough emerged when a study conducted during an outbreak in Diglipur in North Andaman uncovered compelling evidence of a leptospiral origin for AHF. This marked a significant turning point and the first documented occurrence of severe pulmonary haemorrhage as a complication of leptospirosis in India. Since then, numerous outbreaks and sporadic cases of this ailment have come to light from different parts of the country, such as Surat, Cochin, Orissa, Mumbai, and Chennai^21^.

In all these cases, the etiological agent was *Leptospira*. From 1988–2005 surveillance system of Andaman & Nicobar reported 1126 cases. Gujarat reported 3121 cases with 383 deaths from 1997–2005. Chennai, Tamilnadu reported 5452 cases in the time interval of 2004–2006. From 1998–2005, Maharashtra reported 4484 leptospirosis cases with 394 deaths. Besides the unawareness and underreported cases of leptospirosis, other states of India, namely, Punjab, Haryana, Himachal Pradesh, and Karnataka also reported cases of leptospirosis. Numerous outbreaks of leptospirosis were reported after flooding and cyclones in urban areas of the Country. It is significantly overgrown in central, southern, western, and eastern India, where animal rearing practices, unplanned urbanization, rural way of life, and heavy monsoon all contribute to its spread. Specifically, every year, the number of cases is highest between July and October. The outbreak of leptospirosis has been rising in India for the last three decades. The burden of leptospirosis is higher in India due to poor sanitation, lack of diagnosis, and obliviousness among the human population. To overcome these shortcomings and to address the rising burden of leptospirosis, the government of India started a project on the prevention and control of leptospirosis^22^. The positivity rate for the infection is recognized in the southern part of India at $25.6\%$, heeded by $3.5\%$, $3.3\%$, $3.1\%$, and $8.3\%$ in western, central, eastern, and northern India, respectively. In India, leptospirosis is endemic in 5 states and one union territory of India. The endemic states are Gujarat, Maharashtra, Kerala, Tamil Nadu, Karnataka, and the union territory of Andaman & Nicobar Islands. In endemic states, the Government of India has issued treatment guidelines for leptospirosis under the prevention and control program to control leptospirosis in endemic states^22^.

Infectious disease dynamics are also studied using mathematical models. They can provide a quantitative description of the intricate, nonlinear disease transmission process, and they are a simplification of reality that aids in making public health policy decisions. Through this process, various leptospirosis dynamics models have been created using a system of differential equations that considers various species and population structures. For rodents that are primary reservoir hosts for leptospirosis, the dynamics of the infection have been modeled^23^. A good number of literature is available, conducting to comprehend the dynamics of leptospirosis infection in humans, rats, and free-living bacteria^24^, and some models explain how the disease is transmitted from rodents to the human population^25–28^. Some models account for the transmission of leptospirosis in livestock cattle infection^29^. In Tanzania, Holt et al. proposed a mathematical model for the dynamics of leptospirosis^30^ and concluded that eliminating the reservoir rodents reduces the number of human leptospirosis cases. A deterministic mathematical model for leptospirosis disease transmission was evaluated by Triampo et al.^25^. In their research, they took into account leptospirosis cases in Thailand and established their numerical simulations. Pongsumpun^26^ developed another mathematical model to investigate the behavior of leptospirosis infection. He included rodent and human populations in his research and divided the latter into two sub-populations: juveniles and adults. Zaman et al.^27^ demonstrated the dynamic interaction between infected animals and the human population. Further, their work included bifurcation analysis and numerical simulation for various infection rate values.

Unlike the above described, Carrasco and Olmos^24^ considered that humans could be infected by many animals that serve as a reservoir for the bacteria and proposed some techniques for controlling leptospirosis infection. Minter et al.^31^ showed that the environment plays a vital role in transmitting leptospirosis. In contrast to earlier models that account for exponential growth, Gallego and Simoy^32^ studied the dynamics of the zoonotic disease leptospirosis in rodents and the human population by considering rodents’ logistic growth. In their work, they demonstrated the numerical simulations and quantified the impact of control measures on infection dynamics. Chong et al.^33^ presented a compartmental model formulated by Pongsumpun^26^ and Triampo et al.^25^ for leptospirosis in Malaysia. According to their research, a significant factor in determining the number of infected humans is the rate of transmission from susceptible to infected animals and the birth of reservoir animals. Additionally, they demonstrated how their model could forecast the outbreaks observed in Malaysia.

In this paper, we capture the dynamics of leptospirosis in India through mathematical modeling, where we have advanced the modeling of leptospirosis by incorporating a crucial element that distinguishes it from previous studies: the temperature-dependent square growth rate of *Leptospira*, the causative bacteria of leptospirosis. Recognizing the profound influence of environmental temperature on the bacteria’s growth rate and the infection process, we have harnessed this critical factor to refine our model. This enhancement allows us to more accurately capture leptospirosis transmission dynamics, as it accounts for the intricate relationship between temperature and infection rates. By integrating this novel feature, our model offers a deeper understanding of the disease’s behavior and a more robust tool for studying its epidemiology and control strategies. Section 2 informs about the material and methods, namely, the study area Kerala (one of the states of India), the surveillance data obtained from the Government of Kerala^34,35^ and the detailed description of model formulation. Section 3 gives the details of how the system parameters of the proposed model are being estimated, along with their sensitivity analysis and subset selection. Numerical results and their interpretation with respect to the spread of leptospirosis are discussed in Section 4. The paper concludes by providing some specific and actionable policy recommendations to control the spread of leptospirosis in India.

**Figure 1:**
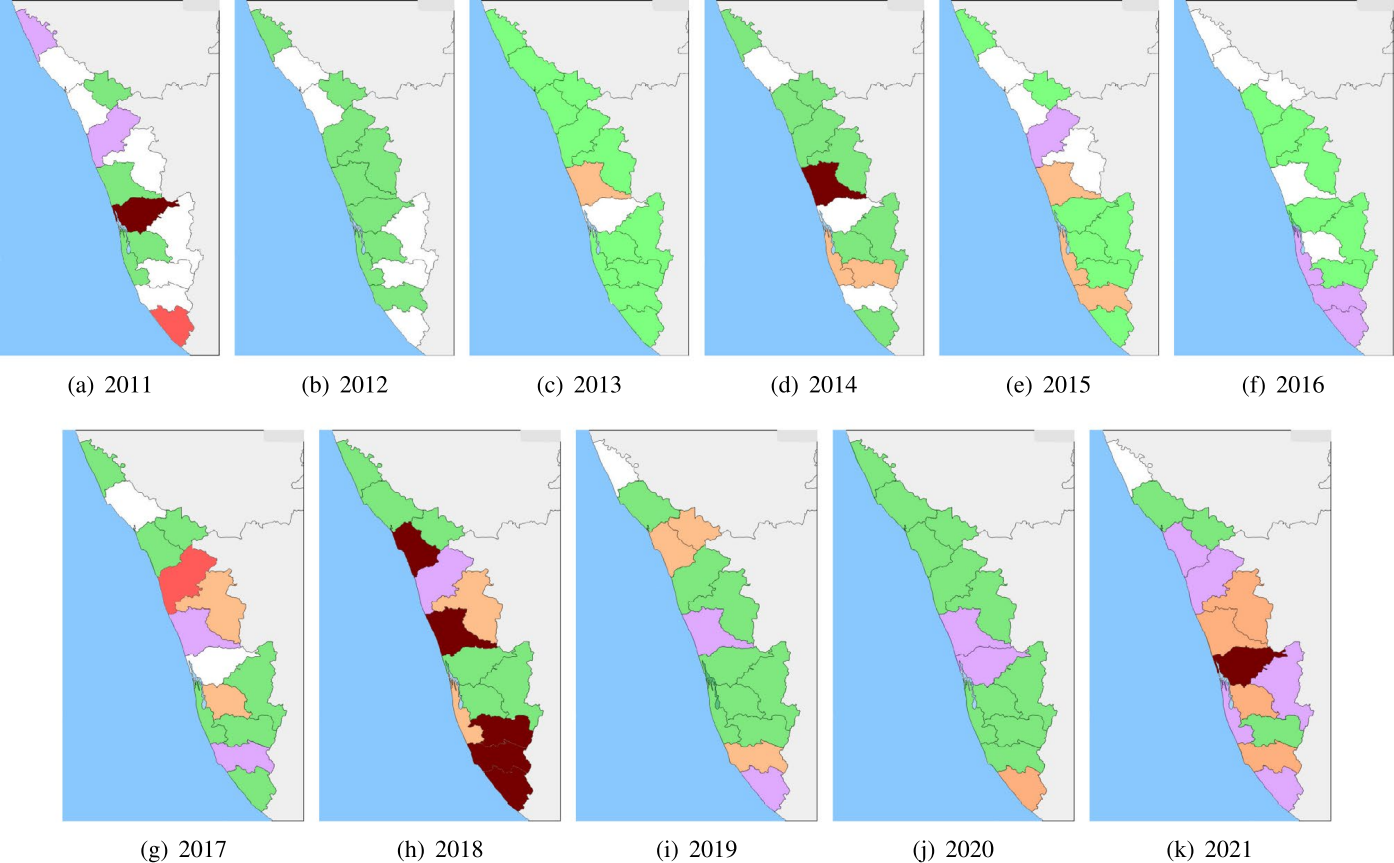
The map illustrates the district-wise incidences of leptospirosis in Kerala, India, over 11 years from 2011 to 2021. The color scheme used highlights the severity of the disease burden, with red indicating incidences above 25, brick red indicating deaths above 10, pink denoting deaths between 8 and 10, orange denoting 5 to 7 deaths, green denoting the number of incidences between 1 and 4, and white indicating no incidence or no record..

## Material and methods

### Study area

India’s Malabar Coast includes the state of Kerala. According to area and population, it is India’s $13$th most populous and $21$st most significant state. Thiruvananthapuram is the capital of the state, with 14 districts. Kerala had 33 million people living there, spread over 38,863 km^2^ per the most recent census in 2011^36^. The state’s coastline stretches for 595 kilometers, and 1.1 million people depend on the fishing business, which contributes $1.58\%$ to the total GDP^36^. It is the second-most urbanized significant state in India, with a $47.7\%$ urban population, according to the 2011 census^36^.

Kerala has a tropical wet climate that varies from district to district. The average annual rainfall in Kerala is 2923 mm. While some of Kerala’s arid lowland areas only receive 1250 mm on average, the mountains of the Idduki district acquire more than 5000 mm of orographic precipitation: the most elevated in the state^36^. Its economy is broad; the tertiary sector, which accounts for the majority of Kerala’s GDP ($64.6\%$), is followed by the secondary sector (contributes $24.79\%$) and the primary sector (contributes $10.45\%$), which includes agriculture, forest, fishing, mining, and quarrying^36^.

### Surveillance data

Surveillance data on leptospirosis from 2005 to 2021 is retrieved from the State Surveillance Unit, Directorate of Health Service, Department of Health and Family Welfare Government of Kerala^34,35^. To enhance and intensify India’s leptospirosis response and surveillance system, the Government of India prompted Integrated Disease Surveillance Project (IDSP) in November 2004 with aid from the World Bank. It intends to detect early stages of leptospirosis, give warning of approaching outbreaks, and initiate adequate responses. This program strengthened the public health laboratories and developed human resources, which includes training of the district surveillance officers, rapid response teams, state surveillance officers, health workers, and other medical and paramedical staff. The IDSP depicts a suspected case of leptospirosis as an individual with body aches, headache, fever, and may have a dry cough, sore throat, diarrhea, dysuria with either (i) possible exposure to occupational or environmental hazards or (ii) existence of the one or more of the following mentioned symptoms and sign like jaundice, cardiac failure, convulsion, and coma. Case investigations are conducted by local authorities weekly and then relaid to the state level and national levels.

For this study, we have considered those cases of leptospirosis demonstrated by the health administration^34^. From 2005 to 2021, there were 21,215 leptospirosis cases in Kerala, India. We have plotted the annual incidence of humans (Fig. S1a), which ranges from $5.3\%$ in 2005 to $16.9\%$ in 2007. After 2007, the incidence rate declined till 2012 ($2.4\%$), then slightly increased in 2013 ($4.2\%$), and again decreased till 2016 ($2\%$). After 2016, the incidence rate due to leptospirosis increased, and in 2021, it was $5.6\%$, with 97 deaths. The monthly trend of the number of leptospirosis cases obtained from the Department of Health and Family Welfare Government of Kerala^34^ is also plotted (Fig. S1b–d). We observe that the number of leptospirosis cases was higher between July and November, with the highest value in August and September, on an average. We also observe the spatial variation and find that the incidence rate is higher in the coastal districts of Kerala, India, compared to the other sections (Fig.1).

### Model formulation

Leptospirosis, caused by the bacterium *Leptospira interrogans*, is carried by animals (namely, rodents, farm animals, and dogs), and lives in their kidneys. The bacteria contaminate the water and soil through the urine of the infected animals. Humans coming in contact with contaminated soil or water get infected, the bacteria enter the body through scratches, open wounds, or dry areas. Keeping these dynamics in mind, we propose the schematic diagram (Fig. S2), showing the transmission route of leptospirosis from the infected animal population to susceptible humans. The proposed model is

1 $$\begin{aligned} \begin{aligned} \frac{d S_A}{d t}&=\lambda _1 -\frac{c_1T_d S_A I_A}{S_A+h_1}-d_AS_A,\\ \frac{d I_A}{d t}&= \frac{c_1T_d S_A I_A}{S_A+h_1}- d_A I_A - d_{L_1} I_A,\\ \frac{d S_H}{d t}&=\lambda _2- \frac{c_2 T_d S_H I_A}{S_H+h_2}-d_H S_H +r_H I_H,\\ \frac{d I_H}{d t}&= \frac{c_2 T_d S_H I_A}{S_H+h_2}- d_H I_H - d_{L_2} I_H - r_H I_H, \end{aligned} \end{aligned}$$

subjected to initial conditions

$$\begin{aligned} S_A(0)>0,~ I_A(0)>0,~ S_H(0)>0 \text { and } I_H(0)\ge 0. \end{aligned}$$

Here, $S_A$ is the susceptible animal population density (namely, rats and livestock), and $I_A$ is the infected animal population density. The susceptible animals grow at a constant rate $\lambda _1$ and get infected when they come in contact with infected animals, the interaction term $\displaystyle \frac{c_1T_d I_AS_A}{S_A+h_1}$ follows Holling type-II functional response, where $T_d$ gives the temperature dependent *leptospire* growth rate (see subsection 3.2), $c_1$ is the infection rate when the susceptible animals comes in contact with the infected ones, $h_1$ is the half saturation constant, $d_A$ is the natural death rate of animals (both susceptible and infected), and $d_{L_1}$ is the death rate of infected animals due to leptospirosis, with the assumption that animal population never recovers from the disease. The Holling Type-II functional response was the natural choice to capture the intricate interaction dynamics. It accounts for a saturation effect, recognizing that as the density of susceptible hosts increases, the transmission rate may plateau due to reduced opportunities for contact between susceptible and infected individuals^37^. Importantly, our choice aligns with observed epidemiological patterns in leptospirosis outbreaks. Not every susceptible host succumbs to the disease and this model faithfully reflects that reality. In a similar manner, we define $S_H$ and $I_H$ to be the susceptible and infected human population densities, respectively. The susceptible humans grow at a rate $\lambda _2$, and get infected when they come in contact with the infected animals (indirectly, or directly), which explains the term $\displaystyle \frac{c_2T_dI_AS_H}{S_H+h_2}$ in the last two equations, $c_2$ being the infection rate, and $h_2$ is the half saturation constant. $d_H$ is the natural death rate of human population density, and $d_{L_2}$ is the death rate of humans due to leptospirosis. Infected humans recover at the constant rate of $r_H$. In the proposed model, we also assumed that there is no leptospirosis transmission from human to human and$/$or human to an animal; only infected animals can infect susceptible animals and humans.

The model assumes that when infected humans recover, they immediately become susceptible again, implying that the recovery from the infection does not convey any immunity. Current research suggests that post-infection immunity is mainly dependent on the presence of serovar-specific antibodies. These antibodies offer protection against reinfection by the same serovar as long as their concentration remains sufficiently high^38^. However, it is essential to note that this immunity is serovar-specific and may not extend to other serovars.

***Note:*** While our model explicitly incorporates the temperature-dependent square growth rate of *Leptospira*, a primary causative agent of leptospirosis, it is essential to recognize the interconnectedness of various other environmental factors. Rainfall and flooding, as highlighted in the introduction, indeed play pivotal roles in disease transmission. They influence the environment’s temperature, creating a dual impact on leptospirosis dynamics. When rainfall occurs, the environmental temperature typically drops, which affects the growth rate of *Leptospira*, aligning with our temperature-dependent model. Moreover, flooding does not directly increase the growth rate of *Leptospira*; instead, it enhances the transmission of leptospirosis by facilitating the spread of the bacteria. Contaminated water sources become more accessible to susceptible populations during these events, leading to increased exposure routes and a heightened risk of infection. Hence, parameters $c_1$ and $c_2$ indirectly take care of the flooding effect, and we have considered that while estimating the ranges of those two parameters. Our model acknowledges the intricate interplay between temperature, rainfall, and flooding, recognizing that these environmental factors collectively shape the epidemiology of leptospirosis.

## Approaches to the estimation of system parameters

Variability in experimental data presents a significant challenge for accurately estimating parameters associated with biological systems. This obstacle is one of the most complex problems that modelers encounter, necessitating a range of approaches to estimate relevant model parameters. Among these, sensitivity analysis stands out as a powerful tool that simplifies parameter estimation by breaking it down into more manageable stages. By using sensitivity analysis, we can categorize less sensitive and unidentifiable parameters, thereby reducing the set of highly sensitive parameters to a more tractable size, which can then be estimated from the available experimental data.

In developing physiologically based mathematical models, it is often essential to estimate parameters from data, as these parameters typically have biological relevance. However, when these parameters are insensitive, estimating them can be challenging despite their significance. Sensitivity analysis provides critical insight into which state outputs are sensitive to given parameters and whether we can expect to estimate the parameters from available data. By understanding which model parameters most influence the model, we can develop more effective models that better represent biological systems.

### The sensitivity analysis and subset selection

The process of sensitivity analysis plays a crucial role in identifying which state outputs are affected by particular model parameters and determining the parameters that significantly influence the model. Additionally, sensitivity analysis evaluates the feasibility of estimating parameters from the available data through subset selection. In order to address the correlation between parameters, the sensitivity function matrix is analyzed. This matrix is used to represent the relationship between the model’s outputs $z=(z_1(t),z_2(t),z_3(t),\ldots ,z_k(t))$ and parameters $g=(g_1,g_2,\ldots ,g_l)^T$ at different time points $t=(t_1,t_2,\ldots ,t_m)$. The matrix is represented as a $km\times l$ matrix as

$$\begin{aligned} P(g,t)= \begin{pmatrix} p_{1,1}(g,t_1) &{}p_{1,2}(g,t_1)&{} . &{} . &{} . &{}p_{1,l}(g,t_1)\\ .&{}.&{}.&{}.&{}.&{}. \\ .&{}.&{}.&{}.&{}.&{}\\ .&{}.&{}.&{}.&{}.&{}.\\ p_{1,1}(g,t_m) &{}p_{1,2}(g,t_m)&{}.&{}.&{}.&{}p_{1,l}(g,t_m) \\ p_{2,1}(g,t_1) &{}p_{2,2}(g,t_1)&{}.&{}.&{}.&{}p_{2,l}(g,t_1) \\ .&{}.&{}.&{}.&{}.&{}. \\ .&{}.&{}.&{}.&{}.&{}. \\ .&{}.&{}.&{}.&{}.&{}. \\ .&{}.&{}.&{}.&{}.&{}. \\ p_{k,1}(g,t_m) &{}p_{k2}(g,t_m)&{}.&{}.&{}.&{}p_{k,l}(g,t_m) \end{pmatrix}, \end{aligned}$$

where $\displaystyle p_{i,j}(g,t_m)= \frac{ \partial z_i(t_m)}{\partial g_j}$ denotes the sensitivity of output $g_j$ at time $t_m$.

**Figure 2:**
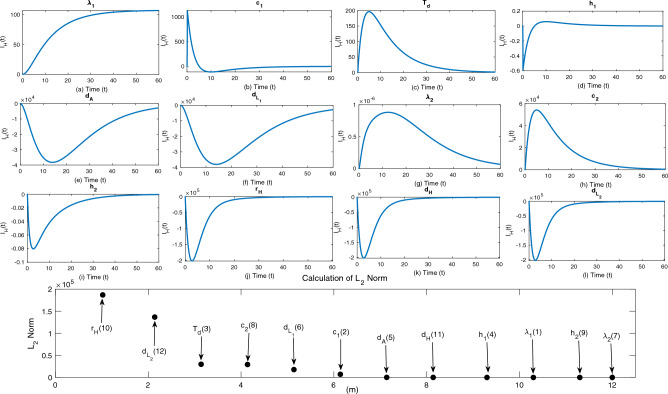
Sensitivity curves and quantification. The observation window is [0, 60], and the parameter’s sensitivity is identified by the maximum deviation of the state variable (along the y-axis). It also determines the time intervals during which the system is most sensitive to such changes. Figures (a-l) show sensitivity curves of the 12 system parameters, and figure m shows the quantification of the sensitivity coefficients using the $L^2$-norm.

We have twelve parameters in our model for which we need to evaluate the sensitivities and rank the parameters according to their identifiability. To obtain the sensitivity graph, we use the code **myAD**^39^ developed by Martin Fink. The sensitivity curves are seen in Fig. 2(a–l). In order to quantify the sensitivity coefficient for each parameter in the model, we first non-dimensionalize the sensitivity functions. We next compute the $L^2$-norm of the resulting functions, given by

$$\begin{aligned} C_{ij}=\bigg \Vert \frac{\partial x_i}{\partial g_j} \frac{g_i}{\max (x_i)}\bigg \Vert _{L^2}^{2}=\int _{t_0 }^{t_f}\bigg |\frac{\partial x_i}{\partial g_j} \frac{g_i}{\max (x_i)}\bigg |^ 2 dt. \end{aligned}$$

We order the parameters from most to least sensitive (in descending order) after ranking and comparing the sensitivity functions (Fig. 2m). In an ordinary least square sense, the Fisher Information Matrix $F=P^{T}P$, is a first-order estimation of the Hessian of the cost function. Consequently, the parameters *g* can be recognized locally only if the rank of the matrix $P^T$ is equal to *l*, where *l* is the total number of unknown parameters.

In this study, we utilize the automatic differentiation^39^ to compute the normalized sensitivity function matrix *P*. Based on the numerical rank of $P^T P$, which is calculated as 6 in our case (with a square root of machine precision epsilon $\epsilon =10^{-4}$), only 6 parameters are found to be identifiable due to their mostly linearly independent spanning set. To identify a subset of identifiable parameters, we employ the *QR* factorization with column pivoting using the Matlab routine **qr**, namely, $\textbf{qr}(F)=[Q\; R\; P]$. This method results in a permutation matrix *W* such that $FW = QR$, by utilizing a column pivoting strategy. The first *k* columns of *W* represent the most estimable *k* parameters, which are [$r_H$, $d_{L_2}$, $T_d$, $c_2$, $d_{L_1}$, $c_1$] in our case, out of 12 parameters, that are ranked as the most identifiable and sensitive.

### Parameter estimation

Numerous studies suggest that a population’s risk of leptospirosis is influenced by the local weather, namely, tropical and subtropical climates. In temperate climates, the temperature is the limiting factor for *leptospire* survival in the environment. As there are very few mathematical studies on relationships between leptospirosis incidence rate with temperature dependent parameter, we intend to develop a mathematical model that correlates the spread of leptospirosis among human and animal populations, taking the temperature of the place or state into consideration.

The optimal growth temperature for pathogenic *leptospires* is 28–30 °C^40^. We use Ratkowsky’s nonlinear regression model^41^ to simulate *leptospire’s* temperature dependence growth, namely,

2 $$\begin{aligned} T_d=\sqrt{G}=\alpha \left( T-T_{\min }\right) ~\left( 1-e^{\beta \left( T-T_{\max }\right) }\right) , \end{aligned}$$

where *G* is the temperature dependent growth rate of *leptospire*, $\alpha$ and $\beta$ are the slopes of the regression curve, which could be used as constants in Eq. (2), $T_{\min }$ and $T_{\max }$ are the minimum and maximum temperature, respectively, at which the growth rate of *leptospire* is zero. Here, *T* is the average temperature of a particular month.

To estimate the values of parameters $\alpha$ and $\beta$ of the temperature dependent function (2), we use the least-square method. We obtain four data sets from the bacterial cultures of *Serratia marcescens, Pseudomonas fluorescens, Escherichia coli* and *Pseudomonas aeruginosa*^41^ (Table S1). Their entire biokinetic temperature range is similar to that of *Leptospira*^42^ and their growth dynamics support the square root relationship proposed by Ratkowsky et al.^41^ Using the method of least square, we obtain the curve of best fit (Fig. S3) with estimated values of $\alpha$ and $\beta$ (Table 1). The interval of estimation of $\alpha$ and $\beta$ are respectively, $\displaystyle (2.7893, 3.9988)$ and $\displaystyle ( 0.2776,0.4141)$ (see SI S2.1).

**Table 1:** Estimated parameters values of $\alpha$ and $\beta$.

Estimated parameters	Data set 1	Data set 2	Data set 3	Data set 4
$\alpha$	3.6402	3.6628	3.4264	2.8468
$\beta$	0.3864	0.2895	0.3367	0.3708

We will now estimate the range of the square root growth rate of *Leptospira* by taking into consideration the minimum temperature $7.1$ °C ($T_{min}$) and maximum temperature 34 °C ($T_{max}$), needed for the survival of the bacteria *Leptospira*. The mean values of the estimated parameters $\alpha$ and $\beta$ are 3.3940 and 0.3458, respectively. Over the years, Kerala has temperature ranging between $22^0$C and $32^0$C. The monthly average temperature (T) for Kerala is given in Table S2^43^. Using (2) and the parameter values of $\alpha$, $\beta$, $T_{min}$, $T_{max}$ and T, we obtain 12 values of $T_d$, the square root growth rate of *Leptospira* (Table S3).

**Figure 3:**
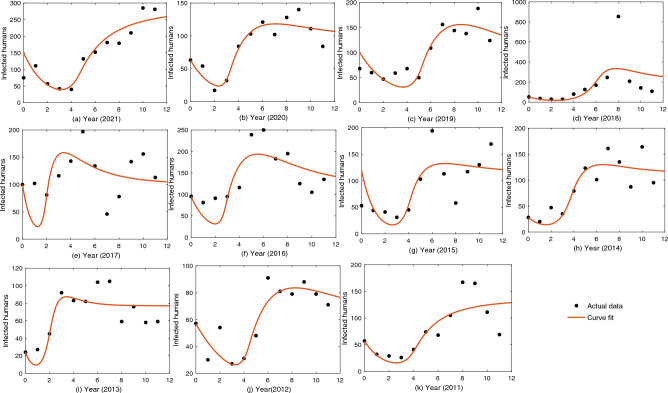
The system parameters, $c_1$, $c_2$, $r_H$, $d_{L_1}$, and $d_{L_2}$, are estimated using the least square method with 11 years of data with 12 data points for each year. The resulting best-fit curves are shown in the figures.

We now want to estimate an interval in which the values of $T_d$ lies. The 12 values of $T_d$ are now considered as a sample of size 12 with an unknown population standard deviation, which will be used for a $95\%$ confidence interval for the mean of $T_d$. Since the population standard deviation is unknown, the sample will follow a t-distribution with ($n-1$), that is, 11 degrees of freedom. The sample mean is $\displaystyle \bar{x}=\frac{\sum \nolimits _{i=1}^{12} x_i}{n} = 60.61$ and the sample standard deviation is $\displaystyle S^2=\frac{\sum \nolimits _{i=1}^{12}(x_i-\bar{x})^2}{n-1} = 1.2$. Hence, the $95\%$ confidence interval is $\displaystyle \left( \bar{x}- t_{0.025,11}\frac{S}{\sqrt{12}},~ \bar{x}+ t_{0.025,11}\frac{S}{\sqrt{12}}\right) = (59.91, 61.30)$, where $t_{0.025,11}$= 2.201 (from the t-distribution table).

We next estimate the parameters $d_A$, $d_H$, $h_1$, and $h_2$. In India, the average life span of animals (rats, pigs, and cattle) varies from 0.4 to 20 years, and that of humans as  (60–70) years^44^. Therefore, we consider the range of natural death rate of animals ($d_A$) to be [0.0042, 0.21] and that of humans($d_H$) to be [0.00119, 0.001389] (the years are converted into months, and then, their reciprocals give the death rates). The values of $\lambda _1$ and $\lambda _2$ are taken as 20,000 and 500 respectively^34^. We assign the values 10,000 and 2,500,000 to $h_1$ and $h_2$ as half saturation population constants. These values are determined based on the 2011 census data, which reveals that Kerala had a total agricultural population of 1653601, with 740403 individuals actively engaged in cultivation. Furthermore, the human population involved in various other activities amounted to 7,532,484 individuals. Considering these figures, a conventional estimate suggests that approximately 5,000,000 individuals^45^ (without loss of generality) participate in occupations such as rice farming, fishing, and other activities with a significant risk of leptospirosis infection^45^.

To estimate the sensitive parameters $c_1$, $c_2$, $d_{L_1}$, $d_{L_2}$, and $r_H$, we use the official reports of the State Surveillance Unit, Directorate of Health Service, Department of Health and Family Welfare, Government of Kerala^34^, regarding the number of infected humans in Kerala due to leptospirosis, from 2011 to 2021. The estimation method starts with the arbitrary selection of initial values for the parameters within the biologically meaningful range. Using the reported data, the initial condition for infected humans can be defined, and thus $I_H(0)$ is considered the same as the data of January for each year at the beginning of the time series. However, since there is no information about infected animals in the reported data, we assume a hypothetical value for $I_A(0)$. The unknown parameters, such as the transmission rate of leptospirosis among animals ($c_1$), the transmission rate of leptospirosis from infected animals to the human population ($c_2$), the rate of recovery of humans ($r_H$), the death rate of animals due to leptospirosis ($d_{L_1}$), and the death rate of infected humans due to leptospirosis ($d_{L_2}$), are obtained by minimizing the error between the fitted curve and the actual data using the least square method. The estimated values of $c_1$, $c_2$, $r_H$, $d_{L_1}$ and $d_{L_2}$ are listed in Table S4. Figures 3(a–k ) show the best fit estimates for the model parameters $c_1$, $c_2$, $r_H$, $d_{L_1}$, and $d_{L_2}$, for all 11 years, namely, 2011–2021. As we have 11 estimated values of each parameter, we obtain a $95\%$ confidence interval for each of the estimated parameters, which follows a t-distribution with 10 degrees of freedom (for details, see supplementary information). The confidence intervals of the estimated parameters are presented in Table 2.

**Table 2:** Definition of parameters used in the model, their units, values and sources.

Parameters	Description	Units	Values	Source
$\lambda _1$	Constant input of susceptible animals	per time	20000	^34^
$\lambda _2$	Constant input of susceptible humans	per time	500	^34^
$c_1$	Infection rate among animals	per time	[0.0305, 0.0496]	Estimated
$c_2$	Infection rate between animals and humans	per time	[9.633$\times 10^{-5}$, 3.9822 $\times 10^{-4}$]	Estimated
$h_1$	Half saturation population of animals	No. of individuals	10000	^45^
$h_2$	Half saturation population of humans	No. of individuals	2500000	^45^
$d_A$	Rate of natural death of the animals	per time	[0.0042, 0.21]	Estimated
$d_H$	Rate of natural death of the humans	per time	[0.00119, 0.001389]	Estimated
$d_{L_1}$	Death rate of animals due to leptospirosis	per time	[0.1457, 0.3387]	Estimated
$d_{L_2}$	Death rate of humans due to leptospirosis	per time	[0.0372, 0.4821	Estimated
$r_H$	Recovery rate of humans	per time	[0.3402, 0.7191]	Estimated
$T_d$	Temperature dependent growth rate of *leptospire*	—–	[59.91, 61.30]	Estimated

## Numerical results and discussion

**Figure 4:**
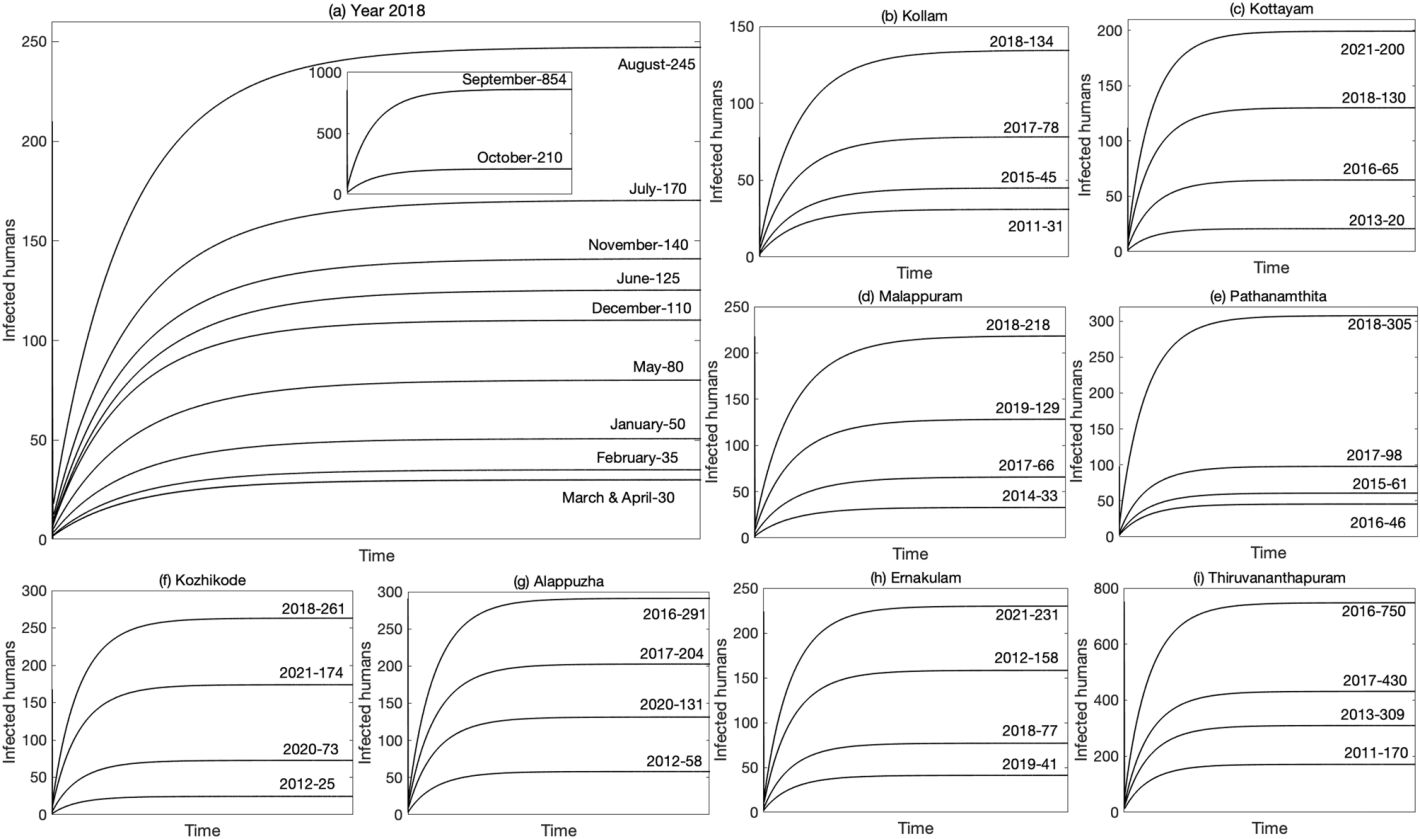
(**a**) shows the predicted monthly number of leptospirosis cases in Kerala, India, for the year 2018. Figures (a–i) represent the district-wise predicted number of leptospirosis cases in Kerala, India, from 2011 to 2021, which offer insightful information regarding the disease’s potential trends and patterns.

We now validate the actual data of the leptospirosis spread in various states of India, namely, Kerala, Gujarat, Karnataka, Maharashtra, and Tamil Nadu, with the simulation results of the proposed mathematical model. Figure 4a shows the monthly dynamics of leptospirosis for the year 2018 in Kerala, namely, the number of infected cases in each month for the year 2018. The predicted values by our model match with data of the State Surveillance Unit, Directorate of Health Service, Department of Health and Family Welfare, Government of Kerala^34^ (Table S5). From figure 4a (inset), it is observed that the maximum number of infected cases was 854, in the month of September 2018, which is 608 cases more than the previous month. The sudden surge in the number of leptospirosis cases is the result of a catastrophic flood during August and September 2018 due to unusually heavy rainfall^35^. One of the most affected district of Kerala, Kozhikode (261), reported the highest number of leptospirosis cases (commonly known as rat fever) and became a major threat to the outbreak of leptospirosis in Kerala. In fact, all the districts of Kerala (Kollam, Kottayam, Malappuram, Pathanamthitta, and Kozhikode), which had direct contact with the flood water, showed a sudden increase in leptospirosis cases^46^ (Figs. 4(b–f) and S4).

**Figure 5:**
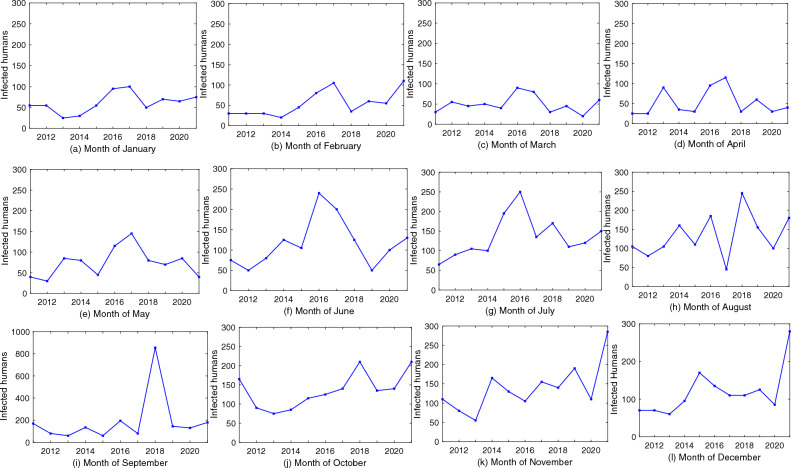
The figures show the monthly trends in the number of leptospirosis cases in Kerala, India, from 2011 to 2021, which has been plotted from the predicted values.

Let us now look into the 11 years scenario of the dynamics of leptospirosis in Kerala. Figures 4a and S5–S14 give the predicted number of infected cases of leptospirosis month-wise in a particular year, namely, from 2011 to 2021 (Table S5). This predicted number matches with the report of Department of Health and Family Welfare, Government of Kerala^34^. We now plot (Figs. S5–S14) the monthly prediction of leptospirosis cases from 2011 to 2021 (11 years), to obtain a trend of the leptospirosis dynamics in Kerala (Fig. 5). It is observed that the number of cases varies between 50 and 100 (Fig. 5(a–d)) in the month of January, February, March, and April for 11 years (2011–2021). In the years 2015, 2016, and 2017, there was an increase in the number of infected cases during the months of January–April. January remains cooler in Kerala with comparatively dry days, moving towards the hottest and driest month April^34^. Due to the cool dry weather, the growth of leptospirosis is subdued, and therefore, the infected number is restricted below 100. However, the number is not reduced to zero (disease free equilibrium) due to occupational activities. People are busy with harvesting at the end part of January and sowing for the next crop in the first half of February. Intermittent rain during this period with repeated flooding of rat nests are the reasons for the contamination of surface water and paddy fields^47^. It is hazardous to do wetland farming during this period. Pineapple orchard workers are also found to be seropositive to leptospirosis during this period as their working activity leads to scratches in the exposed skin, which form a point of entry for *Leptospira*, the bacteria which causes leptospirosis^47^. Since the first rainy season starts from the month of June in Kerala, the graphs show a rise in the number of cases of leptospirosis from June to September, followed by the second rainy season from mid October to mid November (Fig. 5f–k). Rice, being the principal crop of cultivation and is grown under rain fed conditions during June–August to October–December, there is a close interaction of soil, animals, and humans. From the predicted values (Figs. S5–S14) and the trend (Fig. 5f–l), it is observed that a maximum surge in the cases of leptospirosis occurs during this period.

The graphical representation in  Fig. 4(b–i) also vividly captures the dynamic nature of leptospirosis in Kerala’s various districts over the past decade (2011–2021). Our model’s predictions align perfectly with the actual data^34^, highlighting the accuracy of our analysis (Table S6). The numbers of infected cases in Kozhikode, Alappuzha, Ernakulam, and Thiruvananthapuram are particularly alarming, as they remain significantly high (Fig.4(f–i)). Figure S4 gives the trend of the number of infected cases reported in Alappuzha and Thiruvananthapuram districts, which fluctuates between 90 and 750 cases over an 11-year period (2011–2021). These districts are located along the coast of Kerala, accounting for (13–14) % of the total coastline, respectively, placing them at greater risk for leptospirosis. Coastal regions are recognized to have a higher incidence of leptospirosis due to the existence of water bodies such as lakes, rivers, and canals, which can be infected with the urine of contaminated animals. Furthermore, coastal areas are home to a larger population of rodents, which are the primary carriers of the bacteria that cause leptospirosis^48^. In addition to their coastal location, Alappuzha and Thiruvananthapuram districts are recognized for their substantial cultivation of rice and paddy farming, which demands a significant quantity of water. The stagnant water in the rice fields provides an ideal environment for rat breeding, raising the likelihood of contamination. The utilization of fertilizers and pesticides in rice farming can also facilitate the growth of bacteria, intensifying the risk of infection. Moreover, fishing practices in these districts also play a role in the dissemination of leptospirosis. Fishermen are regularly exposed to polluted water and soil, and they may encounter infected rats or fish that have consumed contaminated water^49^. This elevates their chances of contracting the infection.

The data reveals that Thiruvananthapuram saw a surge in the incidence of leptospirosis cases in 2016 (Fig. S4). The cause of this spike was linked to heavy rainfall, which resulted in augmented exposure to polluted water and soil. Additionally, the absence of knowledge and preventative measures during this period may have played a role in the outbreak. Despite various efforts, the number of human infections in all districts of Kerala has not reached the zero equilibrium point, primarily due to the occupational activities in each district. The cultivation of rice, paddy farming, rubber cultivation, and cattle farming are prevalent in all 14 districts of Kerala. The region is constantly besieged by unforgiving rain that floods rat nests as well as taints surface water and rice paddies across all districts, thereby rendering wetland farming perilous. To compound the danger, laborers engaged in tending to pineapple plantations face the looming threat of contracting leptospirosis. The mere act of working leaves their skin exposed and vulnerable, providing a gateway for the lethal *Leptospira* bacteria to enter their bodies and unleash a devastating infection^47^. The gravity of these hazards cannot be overstated, posing a grave risk to both the environment and human life.

Furthermore, the problem is aggravated by the substantial rainfall in Kerala, particularly during the monsoon season, as discussed above, which produces more stagnant water in fields and raises the likelihood of contamination. The heavy rainfall can also result in flooding and landslides, displacing rats from their habitats and driving them to seek refuge in human settlements, escalating the risk of infection. Apart from agricultural practices and rainfall, the temperature in Kerala is also a contributing factor in the transmission of leptospirosis. The warm and humid climate provides the perfect environment for the bacteria to grow and flourish. The bacteria can survive for extended periods in warm and damp surroundings, such as wet soil and stagnant water, which are prevalent in the state^7^. To sum up, the elevated prevalence of leptospirosis in particular districts of Kerala is a result of multiple factors, which encompass agricultural practices such as rice cultivation, pineapple and rubber plantations, substantial rainfall, and the warm and humid climate^47^.

Occupational hazards, which include rice cultivation, rubber, and pineapple cultivation by farmers, agricultural laborers, and veterinary practitioners, are the main reason for the spread of leptospirosis in the period June- December and in all districts of Kerala. Other reasons are washing clothes in canals and ponds, home gardening, and contact with domestic pets. Because of the availability of edible crops like rice and pineapple throughout the year, there is a huge increase in the rodent and cattle population, which leads to frequent contamination of the fields with animal urine^50^. This facilitates the survival rate of *Leptospira*, which remains alive in moist, slightly acidic to alkaline soil even up to 279 days without losing its pathogenicity^47^, resulting in the increased number of human leptospirosis. It should be noted that the unusual spike to the margin of 800 plus cases in 2018 (Fig. 4a) and 700 plus in 2016 in Thiruvananthapuram (Fig. S4) was the result of a catastrophic flood due to unusually heavy rainfall. From the trend (Fig. 5), it is also observed that there is a steady increase in the months of October–December from 2011 to 2021 (11 years). Since Kerala has two rainy seasons, it prevents the soil from drying out. Once again, the increase in the incidence of leptospirosis points toward the seasons and occupational activities, which include the cultivation of three crops of rice in one year. The unprecedented increase in the incidence from October to December in 11 years (2011–2021) is the result of the rice farming season.

**Figure 6:**
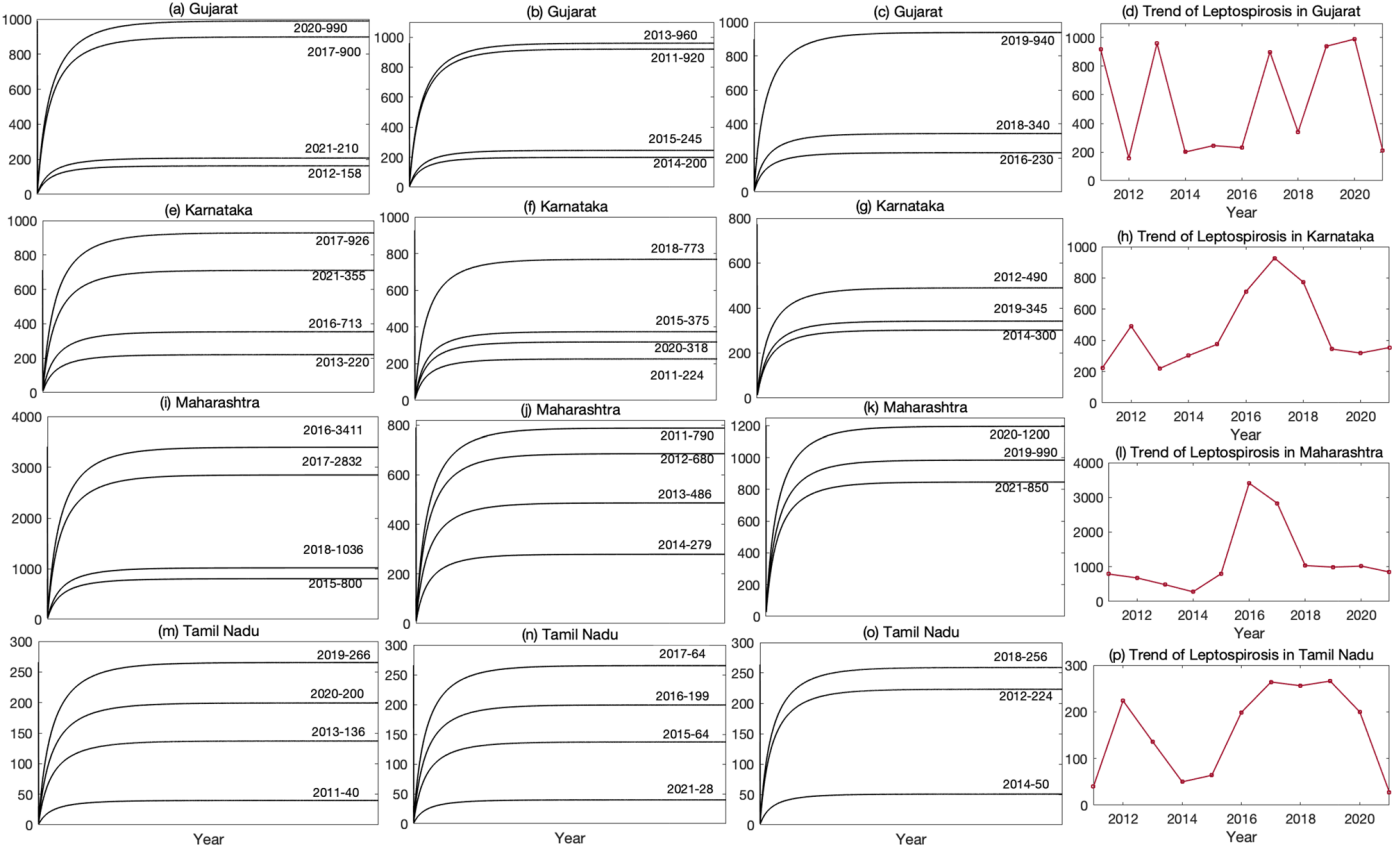
The figure represents the number of leptospirosis cases in different states of India year-wise and their trends.

Figures 6(a–c) show the yearly infection of leptospirosis in humans in Gujarat obtained from the predicted values of the model simulations from the year 2011 to 2021 (Table S7). The trend (Fig. 6d) indicates a higher incidence of the disease in areas of Gujarat, namely, certain areas of South Gujarat where heavy rainfall is common, specifically in districts such as Surat, Navsari, Valsad, and Tapi^51^. South Gujarat is also known for its agriculture and fishing industry, which contributes significantly to the region’s economic growth and development. However, the area’s ideal breeding grounds for rodents, like creeks, marshy lands, and mangrove forests, make it susceptible to the spread of leptospirosis. Fishermen, who play a critical role in the thriving fishing industry, are particularly vulnerable to infection due to frequent exposure to contaminated water sources. *Leptospira* bacteria, which cause leptospirosis, can survive for months in stagnant water and are often found in the urine of infected animals such as rats, dogs, and cattle. In addition to exposure to contaminated water, fishermen in Gujarat are also at risk due to handling fish or seafood contaminated with the bacteria and the presence of cuts and wounds on their hands and feet, which are common in this line of work. Poor sanitation facilities and living conditions further increase the risk of infection. Without access to clean water for washing and bathing, and living in proximity to animals carrying the bacteria, fishermen in Gujarat are particularly vulnerable to leptospirosis. Other factors involved in the preponderance of South Gujarat are paddy cultivation and sugarcane, work practice by the farmer and animal handler, climatic conditions, clay soil structure heads to water logging high water tablets, and rodent carriers^52^. The disease has been endemic in South Gujarat since 1994.

In a similar manner, we obtain the yearly infection of leptospirosis in humans and the corresponding trends for the states Karnataka (Fig. 6(e–h)), Maharashtra (Fig. 6(i–l)), and Tamil Nadu (Fig. 6(m–p)) in the span of 10 years (Table S7). The patterns of leptospirosis infections in humans in these states occur due to a combination of various factors. The trends observed in Karnataka (Fig. 6(e–h)) align with the factors that contribute to human leptospirosis. Incessant rainfall leads to sporadic flooding in low-lying regions, resulting in infrastructural damage. The sources of water, such as ponds, streams, rivers, and canals, become contaminated due to the constant flushing out of forests and farmlands, including rodent burrows. Leptospirosis poses a significant threat to the rural population in Karnataka, which relies on agriculture for sustenance. Waterlogged fields, animal husbandry practices, and communal pond usage by both humans and animals all add to the disease’s propagation^53^.

In Maharashtra, the disease prospers in places with inadequate sanitation and hygiene, as well as in regions with copious rainfall and flooding. The vibrant city of Mumbai is a perfect illustration of this phenomenon, where slum areas are notorious for being cramped, lacking proper waste management, and having substandard sanitation facilities. These slums create an ideal environment for rodents, which are the primary carriers of the *Leptospira* bacteria^54^. The dense population in these areas elevates the probability of human exposure to contaminated water, soil, and food, thereby amplifying the disease’s transmission. The monsoon season in Mumbai heightens the probability of leptospirosis transmission, as intense rainfall results in flooding and water-logging throughout the city. The standing water in the slum areas presents an ideal environment for rodents and other disease-carrying organisms to breed. Additionally, this polluted water can seep into the water supply system, intensifying the risk of infection for the entire population.

Leptospirosis has long been a major health concern in Tamil Nadu, with agriculture practices and other factors similar to those in neighboring Kerala often identified as primary culprits. However, mines are another significant factor, often overlooked, driving the spread of this disease. The blue metal mines in Tamil Nadu are not only a source of construction materials and economic growth, but also a breeding ground for leptospirosis, a disease that has plagued the state^55^. The mining process, which involves excavating large amounts of soil and rock, creates stagnant water, providing rodents with a perfect environment for breeding. These rodents carry the *Leptospira* bacteria, which they spread through their urine and feces, contaminating the water, soil, and other surfaces in and around the mining areas. Unfortunately, those who work or live near the mines are particularly vulnerable to infection. The contaminated water and soil in and around the mining areas serve as a major source of leptospirosis infections, putting people at risk of contracting the disease. This is particularly concerning for those living in rural areas, and rely on agriculture and animal husbandry for their livelihoods, as the mines are often located in these regions. It is crucial to address the issue of leptospirosis in and around mining areas to prevent the further spread of the disease.

Improving waste management practices and increasing awareness of the dangers of leptospirosis can go a long way in reducing the risk of infection. In addition, protective measures such as using gloves, boots, and other protective gear can help those, who work in and around the mines to reduce their risk of infection. It is essential that we tackle these challenges by enhancing infrastructure, waste management, and public awareness campaigns to curb the transmission of leptospirosis in our communities.

## Conclusion

Leptospirosis, a zoonotic disease linked to meteorological variables, has been studied through mathematical modeling using Kerala infection data^34^ to illustrate how these variables affect disease occurrence in tropical settings. In order to anticipate leptospirosis outbreaks, these openly accessible data could be usefully included in the planning process for public health. Consequently, this study aimed to identify the meteorological factors influencing leptospirosis incidence and assess a forecasting model for India’s tropical and subtropical regions. The results of this study highlight the importance of understanding the specific factors contributing to the transmission of leptospirosis in different regions of India. This information can be used to develop targeted control strategies to reduce the disease burden in these regions.

The study is conducted in three sections. The first section focused on the material and methods, including data collection and processing, parameter estimation for the temperature-dependent function, and model construction, showing the dynamics of leptospirosis between humans and animals. The second section presents the analytic results, including the parameter estimations of model parameters using Kerala’s leptospirosis infection data from 2011 to 2021, their confidence intervals, and the model’s sensitivity to those parameters. The sensitivity analysis demonstrates that the model is most sensitive to the values of the transmission rates ($c_1$, and $c_2$), temperature dependent function ($T_d$), death rates due to leptospirosis ($d_{L_1}$ and $d_{L_2}$), and recovery rate ($r_H$). The subset selection confirms that these parameters can be estimated using the available data. The third section presents the numerical results of the dynamics of leptospirosis, followed by a discussion.

The mathematical model used in the study is a SI model, widely used for studying the spread of infectious diseases. The model is constructed by introducing a temperature-dependent function to the transmission rate parameter, which allowed us to investigate the role of meteorological variables in the transmission of leptospirosis. We have used the meteorological data of Kerala to estimate the transmission rate parameters^34^. The results show that the meteorological factor is essential in the spread of leptospirosis. Kerala’s higher rainfall and other favorable meteorological factors (temperature and humidity) led to increased transmission rates. The parameter estimation technique used in the study effectively estimates the values of the parameters, and the confidence intervals for the estimated parameters are calculated to assess the uncertainty associated with the estimates.

We have performed a numerical simulation and predicted the number of infections from 2011 to 2021 for each month using various values of the temperature-dependent function based on Kerala’s weather. We have also discussed the pattern of leptospirosis transmission in other endemic states of India, namely, Gujarat, Karnataka, Maharashtra, and Tamil Nadu. We find that the main factors contributing to the transmission of leptospirosis in these states are excessive rainfall, natural disasters, paddy farming, sugarcane farming, pineapple orchards, mining, and rice cultivation^5,10,14,21,35,46,47,49,50,54,55^. Excessive rainfall and natural disasters increase the likelihood of contaminated water and soil, while paddy farming, sugarcane farming, pineapple orchards, and rice cultivation provide favorable conditions for the survival and transmission of the bacteria. These factors can lead to an increase in the number of leptospirosis cases in these states. Overall, this study highlights the importance of meteorological factors in transmitting leptospirosis and demonstrates the usefulness of mathematical modeling and parameter estimation techniques in understanding the spread of leptospirosis and developing effective control strategies. The study results have important implications for public health policy and provide a basis for further research. In the discussion, we have emphasized the importance of adopting proper sanitation measures and using personal protective equipment (PPE) to control the spread of leptospirosis in the human population. For example, wearing gloves and barefoot boots during paddy farming and other activities involving stagnant water can reduce the risk of exposure to contaminated soil and water. In fact, a compulsory law can be passed, just like wearing a mask during COVID 19. In addition, improving sanitation practices, such as proper disposal of waste and maintaining clean water sources, can also reduce the risk of transmission. These control measures can help to prevent the spread of leptospirosis and reduce the burden of the disease in endemic regions. The results of this study provide important insights into the transmission dynamics of leptospirosis and can advise public health policies aimed at controlling the spread of the disease in India. By adopting proper sanitation practices and using personal protective equipment, we can help to reduce the incidence of leptospirosis and improve the health and well-being of the population.

To effectively combat the spread of leptospirosis, a comprehensive and multifaceted strategy is imperative. Targeted interventions must take center stage in this battle. The following recommendations have been included to guide policy-makers and stakeholders to provide specific and actionable guidance for addressing the spread of leptospirosis effectively:

(i) *Urban and peri-urban areas:* Engage the local community in rat elimination programs, employing techniques like trapping and rodenticides. Strengthen building protection measures by sealing gaps and openings to block rodent access, with oversight from municipal authorities to verify compliance.
(ii) *High-risk occupations:* Develop tailored occupational safety guidelines, emphasizing disinfection protocols, protective clothing mandates, and precise material handling procedures for workers in high-risk occupations like agriculture, mining, veterinary work, and sewage treatment.
(iii) *Agricultural settings:* Advocate for elevated sanitation standards, emphasizing the regular maintenance of cattle sheds and eradicating stagnant water sources. Promote the widespread adoption of cattle vaccination and the utilization of protective gear, like, rubber boots and waterproof bandages among farmers and individuals exposed to flood-prone regions. This concerted effort ensures improved hygiene and safeguards against potential risks.
(iv) *Post-exposure hygiene:* Promote the immediate washing of hands and feet in the event of potential contact with contaminated environments. This swift response is a vital protective measure against potential risks.
(v) *Routine medical check-ups:* Advocate for regular health screenings for animals and humans residing in leptospirosis-endemic areas, enhancing the potential for early detection and prompt treatment.
(vi) *Public awareness campaigns:* Spread awareness regarding leptospirosis risks and preventative measures through extensive public outreach campaigns, crafting messages that resonate with various cultures and disseminating them across various media channels. This holistic approach equips communities with essential knowledge, empowering them to safeguard their health proactively.
(vii) *Resource allocation:* Prioritize the immediate allocation of resources for rat elimination, improved agricultural sanitation, cattle shed maintenance, cattle vaccinations, health assessments for both animals and humans and the launch of awareness campaigns.
(viii) *Stakeholder coordination:* Develop a balanced synergy among stakeholders, potentially through task force creation, to ensure these crucial initiatives’ resolute and effective implementation. This synchronized effort is pivotal in achieving our shared suggestions.
(ix) *Tailored policies:* Consider the invention of customized policies, including initiatives for urban rat control and stringent water quality standards, to fortify our preventive measures. This strategic approach bolsters our defenses and enhances our preparedness.
(x) *Monitoring and evaluation:* Establish robust monitoring and evaluation systems to gauge the efficacy of these interventions thoroughly. This proactive approach ensures we can measure and optimize our impact effectively.

To sum up, the high prevalence of leptospirosis in tropical parts of India is caused by a combination of factors such as the coastal geography, rice and rubber cultivation, paddy farming, cattle farming, fishing practices, and heavy rainfall. To prevent the spread of the disease, it is recommended to implement measures such as rat eradication programs, better sanitation practices in agricultural areas, regular cleaning of cattle sheds, vaccination of cattle, routine medical check-ups for both animals and humans, and awareness campaigns.

### Supplementary Information

Supplementary Information.

## Data Availability

All data generated or analysed during this study are included in this article (and its supplementary information file).
