# Supplementary material for: Modeling the dynamics of leptospirosis in India
Source: Sci Rep. 2023 Nov 13;13:19791. doi: 10.1038/s41598-023-46326-2 (PMC10643689; doi:10.1038/s41598-023-46326-2)
Supplement: Supplementary file 1 — Supplementary Information. [file 41598_2023_46326_MOESM1_ESM.pdf]

Modeling the dynamics of Leptospirosis in India (Supplementary Information)

Antima[1] **and Sandip Banerjee**[2*]

[1,2]Indian Institute of Technology Roorkee, Department of Mathematics, Roorkee 247667, Uttarakhand, India
[*]Corresponding author

ABSTRACT

The supplementary material contains additional tables, figures and parameter values used to obtain numerical results.

S1 Data and Tables

(a) Serratia Marcescens		(b) Pseudomonas Fluorescens		(c) Escherichia coli		(d) Pseudomonas aeruginosa	
**Temp. (K)**	**Square root of growth rate**	**Temp. (K)**	**Square root of growth rate**	**Temp. (K)**	**Square root of growth rate**	**Temp. (K)**	**Square root of growth rate**
295.26	67.13	292.32	48	293.26	51.84	293.29	53.54
297.19	80.97	293.36	55.45	296.14	76.16	296.31	68.66.53
299.12	91.88	295.43	59.59	299.04	79.36	299.19	83.78
301.05	101.54	297.34	67.45	301.12	89.28	301.28	86.29
303.16	107.41	299.24	71.59	303.21	97.92	303.38	91.65
305.09	110.77	301.31	73.24	305.13	103.36	305.21	96.06
307.19	115.80	303.22	87.72	307.22	107.2	307.30	97.95
309.12	115.80	305.29	96.41	309.14	111.36	309.39	101.73
311.22	111.61	307.37	99.72	311.17	115.52	311.36	104.57
313.16	64.19	309.27	111.31	318.13	99.52	313.32	105.19
		311.18	111.31	319.25	93.44	315.16	105.51
		313.43	107.59	321.33	40	317.25	81.57
		315.67	98.49			318.29	65.19
		317.40	72.83			319.21	30.23
		318.44	45.10				
		319.65	23.59				

Table S1. *The temperature and the corresponding square root of growth rate for the bacteria species (a) Serratia marcescens, (b) Pseudomonas fluorescens, (c) Escherichia coli, and (d) Pseudomonas aeruginosa, obtained from [Fig. 2, 41] by using the online platform PlotDigitizer.*

Average Temp.	Jan	Feb	March	April	May	June	July	August	Sept	Oct	Nov	Dec
$^{\circ}C$	26.5	27.3	28.2	28.1	27.3	25.9	25.5	25.4	25.8	26	26.2	26.2

Table S2. *The table provides a comprehensive overview of the monthly weather conditions in Kerala spanning over three decades, from 1991 to 2021 [43].*

Square root of growth rate	Jan	Feb	March	April	May	June	July	August	Sept	Oct	Nov	Dec
T_d	62.36	63.78	64.79	64.72	63.78	61.06	60.11	59.86	60.83	61.29	61.73	61.73

Table S3. *The square root of the growth rate of leptospira considering temperature conditions in Kerala, calculated using the Ratkowsky model [41].*

Estimated parameters	c_1	c_2	r_H	d_{L_1}	d_{L_2}
2011	0.03363	0.00018	0.14939	0.16300	0.49998
2012	0.03733	0.00003	0.00279	0.39	0.28119
2013	0.06242	0.00059	0.79578	0.5	0.99999
2014	0.03113	0.00029	0.438	0.32684	0.29810
2015	0.03324	0.00027	0.92078	0.13159	0.00022
2016	0.05086	0.0001	0.65	0.091	0.02541
2017	0.06550	0.0001	0.82996	0.25	0.64236
2018	0.02706	0.00073	0.64046	0.35	0.07208
2019	0.02814	0.00004	0.388	0.23	0.00025
2020	0.04648	0.00013	0.47101	0.22988	0.03623
2021	0.02501	0.00026	0.54	0.0023	0.00013

Table S4. *Estimated values of model parameters .*

Year	Data	Month											
		Jan	Feb	Mar	Apr	May	Jun	Jul	Aug	Sep	Oct	Nov	Dec
2011	Government data	57	32	29	26	41	74	68	105	167	165	111	69
	Predicted data	55	30	30	25	40	75	65	105	170	165	110	70
2012	Government data	57	30	54	27	31	48	91	81	79	88	79	71
	Predicted data	55	30	55	25	30	50	90	80	80	90	80	70
2013	Government data	24	27	45	92	83	82	104	105	59	76	58	59
	Predicted data	25	30	45	90	85	80	105	105	60	75	55	60
2014	Government data	28	20	47	35	79	123	101	161	135	87	164	95
	Predicted data	30	20	50	35	80	125	100	160	135	85	165	95
2015	Government data	53	44	41	31	45	103	194	113	58	117	130	169
	Predicted data	55	45	40	30	45	105	195	110	60	115	130	170
2016	Government data	95	81	91	95	116	239	250	183	195	125	105	135
	Predicted data	95	80	90	95	115	240	250	185	195	125	105	135
2017	Government data	100	101	81	116	143	197	134	46	78	141	156	113
	Predicted data	100	105	80	115	145	200	135	45	80	140	155	110
2018	Government data	52	36	29	29	80	126	169	246	854	208	141	108
	Predicted data	50	35	30	30	80	125	170	245	854	210	140	110
2019	Government data	68	60	47	59	68	50	109	156	144	138	188	124
	Predicted data	70	60	45	60	70	50	110	155	145	135	190	125
2020	Government data	63	54	17	32	84	103	121	102	128	140	111	84
	Predicted data	65	55	20	30	85	100	120	100	130	140	110	85
2021	Government data	75	111	57	42	40	132	152	181	179	210	285	281
	Predicted data	75	110	60	40	40	130	150	180	180	210	285	280

Table S5. *Actual data and predicted values of leptospirosis spread in Kerala, India for 2011–2021.*

Year	Data	Districts							
		Kollam	Kottayam	Malappuram	Pathana-mthita	Kozhikode	Alappuzha	Ernakulam	Thiruvan-anthapuram
2011	Government data	31	42	71	22	68	86	224	169
	Predicted data	31	-	-	-	-	-	-	170
2012	Government data	9	34	42	22	25	63	158	193
	Predicted data	-	-	-	-	25	58	158	-
2013	Government data	13	19	15	25	63	70	163	309
	Predicted data	-	20	-	-	-	-	-	309
2014	Government data	20	25	33	49	123	76	136	390
	Predicted data	-	-	33	-	-	-	-	-
2015	Government data	45	27	13	61	137	71	115	435
	Predicted data	45	-	-	61	-	-	-	-
2016	Government data	71	65	28	46	129	291	66	752
	Predicted data	-	65	-	46	-	291	-	750
2017	Government data	78	66	66	98	161	204	96	431
	Predicted data	78	-	66	98	-	204	-	430
2018	Government data	134	130	218	312	261	254	77	275
	Predicted data	134	130	218	305	261	-	77	-
2019	Government data	68	80	129	78	168	188	41	220
	Predicted data	-	-	129	-	-	-	41	-
2020	Government data	48	202	32	91	73	131	65	178
	Predicted data	-	-	-	-	73	131	-	-
2021	Government data	63	202	124	87	174	235	231	251
	Predicted data	-	200	-	-	174	-	231	-

Table S6. *Actual data and predicted values (District wise) of leptospirosis spread in Kerala, India.*

Year	Gujarat		Karnataka		Maharashtra		Tamil Nadu	
	**Government data**	**Predicted data**	**Government data**	**Predicted data**	**Government data**	**Predicted data**	**Government data**	**Predicted data**
2011	919	920	-	224	-	790	41	40
2012	157	158	491	490	680	680	225	224
2013	962	960	220	220	486	487	135	136
2014	-	200	302	300	279	278	51	50
2015	245	245	375	375	66	800	63	64
2016	233	230	712	713	3411	3410	199	200
2017	900	900	927	926	2832	2833	265	266
2018	-	340	773	773	1036	1036	255	256
2019	-	940	344	345	-	990	265	266
2020	990	990	319	318	-	1200	0	200
2021	-	210	354	355	-	850	26	26

Table S7. *Actual data and predicted values of leptospirosis spread in Gujarat, Karnataka, Maharashtra and Tamil Nadu.*

S2 Confidence Interval

S2.1 Confidence interval for α and β

We estimate α and β parameters from data collected from four distinct bacterial strains: *Serratia marcescens, Pseudomonas fluorescens, Escherichia coli* and *Pseudomonas aeruginosa* [41].

We structured our dataset to include four observations for every parameter, assuming a t-distribution for their distribution. Subsequently, we calculated the 95% confidence interval for each parameter by determining the mean ($\bar{z}$) and variance (ξ^2) for each parameter, applying the following formulas: $\left(\bar{z}-t_{0.025,3}\frac{\xi}{\sqrt{4}},\ \bar{z}+t_{0.025,3}\frac{\xi}{\sqrt{4}}\right)$.

As a result, we find the sample mean for α and β at $\bar{\alpha} = 3.3940$ and $\bar{\beta} = 0.3458$,, respectively. We also computed the sample standard deviation for α and β, resulting in ${\xi_1}^2 = 0.1444$ and ${\xi_2}^2 = 0.0018$, respectively.

This estimates 95% confidence intervals for α and β are $(2.7893, 3.9988)$ and $(0.2776, 0.4141)$, respectively, using $t_{0.025,10} = 3.18245$.

S2.2 Confidence interval for c_1, c_2, r_H, d_{L_1}, and d_{L_2}

Based on the leptospirosis data collected from 2011 to 2021, we have obtained 11 estimated values for the parameters, namely, c_1, c_2, r_H, d_{L_1}, and d_{L_2}. To determine the confidence interval for each estimated parameter, we constructed a sample space comprising 11 observations for each parameter. We assumed that the distribution of these parameters follows a t-distribution. Consequently, we estimated the 95% confidence interval by calculating each parameter's mean $\left(\bar{y}=\frac{\sum_{i=1}^{11} y_i}{11}\right)$ and variance $\left(\sigma^2=\frac{\sum_{i=1}^{11}(y_i-\bar{y})^2}{10}\right)$. The 95% confidence interval for each parameter is then obtained using the formulas $\left(\bar{y}-t_{0.025,10}\frac{\sigma}{\sqrt{11}},\ \bar{y}+t_{0.025,10}\frac{\sigma}{\sqrt{11}}\right)$. So, the sample mean of c_1 and c_2 is $\bar{c_1}=\frac{\sum_{i=1}^{11} c_{1i}}{11}=0.0401$ and $\bar{c_2}=\frac{\sum_{i=1}^{11} c_{2i}}{11}= 2.4727^{-4}$, respectively. The sample standard deviation of c_1 and c_2 is ${\sigma_1}^2=\frac{\sum_{i=1}^{11}(c_{1i}-\bar{c_1})^2}{10}=2.0177*10^{-4}$ and ${\sigma_2}^2=\frac{\sum_{i=1}^{11}(c_{2i}-\bar{c_2})^2}{10}=5.0482*10^{-8}$. Hence, the 95% confidence interval for c_1 and c_2 are $\left(\bar{c_1}-t_{0.025,10}\frac{\sigma_1}{\sqrt{11}},\ \bar{c_1}+t_{0.025,10}\frac{\sigma_1}{\sqrt{11}}\right)= (0.0305, 0.0496)$ and $\left(\bar{c_2}-t_{0.025,10}\frac{\sigma_2}{\sqrt{11}},\ \bar{c_2}+t_{0.025,10}\frac{\sigma_2}{\sqrt{11}}\right)=(9.633*10^{-5}, 3.9822*10^{-4})$, where $t_{0.025,10}$= 2.306 (from the t-distribution table). Similarly, the sample mean of r_H, d_{L_1} and d_{L_2} are $\bar{r_H}=0.5297$, $\bar{d_{L_1}}=0.2422$ and $\bar{d_{L_2}}=0.2596$, respectively. The sample standard deviation of r_H, d_{L_1} and d_{L_2} are ${\sigma_3}^2=0.0795$, ${\sigma_4}^2=0.0206$, and ${\sigma_5}^2=0.1096$, respectively. The 95% confidence interval for r_H, d_{L_1} and d_{L_2} is then obtained is $[0.3402, 0.7191]$, $[0.1457, 0.3387]$, and $[0.0372, 0.4821]$, respectively.

S3 Parameter values used in figures (Main and Supplementary)

Figure 4(a) $\lambda_1 = 20000$, $\lambda_2 = 500$, $h_2 = 2500000$, $h_1 = 10000$, $c_1 = 0.0309$, $c_2 = 0.0000972$, $r_H = 0.696$, $d_{L_1} = 0.31$, $d_{L_2} = 0.44$, $d_H = 0.0012$, $d_A = 0.153$, and $T_d = 59.94$ (March and April- 30).

$\lambda_1 = 20000$, $\lambda_2 = 500$, $h_2 = 2500000$, $h_1 = 10000$, $c_1 = 0.0309$, $c_2 = 0.0000972$, $r_H = 0.5$, $d_{L_1} = 0.324$, $d_{L_2} = 0.44$, $d_H = 0.0012$, $d_A = 0.153$, and $T_d = 59.94$ (February- 35).

$\lambda_1 = 20000$, $\lambda_2 = 500$, $h_2 = 2500000$, $h_1 = 10000$, $c_1 = .0309$, $c_2 = 0.000124$, $r_H = 0.38$, $d_{L_1} = 0.324$, $d_{L_2} = 0.44$, $d_H = 0.0012$, $d_A = 0.153$, and $T_d = 59.94$ (January- 50).

$\lambda_1 = 20000$, $\lambda_2 = 500$, $h_2 = 2500000$, $h_1 = 10000$, $c_1 = 0.309$, $c_2 = 0.00098$, $r_H = 0.48$, $d_{L_1} = 0.234$, $d_{L_2} = 0.054$, $d_H = 0.0012$, $d_A = 0.153$, and $T_d = 60.94$ (May- 80).

$\lambda_1 = 20000$, $\lambda_2 = 500$, $h_2 = 2500000$, $h_1 = 10000$, $c_1 = 0.0309$, $c_2 = 0.000162$, $r_H = 0.362$, $d_{L_1} = 0.1534$, $d_{L_2} = 0.44$, $d_H = 0.0012$, $d_A = 0.13$, and $T_d = 59.94$ (December- 110).

$\lambda_1 = 20000$, $\lambda_2 = 500$, $h_2 = 2500000$, $h_1 = 10000$, $c_1 = 0.0309$, $c_2 = 0.0001223$, $r_H = 0.37$, $d_{L_1} = 0.234$, $d_{L_2} = 0.054$, $d_H = 0.0012$, $d_A = 0.153$, and $T_d = 60.94$ (June- 125).

$\lambda_1 = 20000$, $\lambda_2 = 500$, $h_2 = 2500000$, $h_1 = 10000$, $c_1 = 0.039$, $c_2 = 0.000167$, $r_H = 0.46$, $d_{L_1} = 0.234$, $d_{L_2} = 0.054$, $d_H = 0.0012$, $d_A = 0.153$, and $T_d = 60.94$ (November- 140).

$\lambda_1 = 20000$, $\lambda_2 = 500$, $h_2 = 2500000$, $h_1 = 10000$, $c_1 = 0.039$, $c_2 = 0.000167$, $r_H = 0.37$, $d_{L_1} = 0.234$, $d_{L_2} = 0.054$, $d_H = 0.0012$, $d_A = 0.153$, and $T_d = 60.94$ (July- 170).

$\lambda_1 = 20000$, $\lambda_2 = 500$, $h_2 = 2500000$, $h_1 = 10000$, $c_1 = 0.03509$, $c_2 = 0.000254$, $r_H = 0.36$, $d_{L_1} = 0.234$, $d_{L_2} = 0.144$, $d_H = 0.0012$, $d_A = 0.09$, and $T_d = 59.94$ (August- 245).
$\lambda_1 = 20000$, $\lambda_2 = 500$, $h_2 = 2500000$, $h_1 = 10000$, $c_1 = 0.0309$, $c_2 = 0.0002$, $r_H = 0.36$, $d_{L_1} = 0.234$, $d_{L_2} = 0.054$, $d_H = 0.0012$, $d_A = 0.153$, and $T_d = 60.94$ (October- 210).
$\lambda_1 = 20000$, $\lambda_2 = 500$, $h_2 = 2500000$, $h_1 = 10000$, $c_1 = 0.04309$, $c_2 = 0.00038$, $r_H = 0.372$, $d_{L_1} = 0.15$, $d_{L_2} = 0.054$, $d_H = 0.0012$, $d_A = 0.15$, and $T_d = 61.094$ (September- 854).

Figure 4(b) $\lambda_1 = 20000$, $\lambda_2 = 500$, $h_2 = 2500000$, $h_1 = 10000$, $c_1 = 0.0309$, $c_2 = 0.0000972$, $r_H = 0.59$, $d_{L_1} = 0.34$, $d_{L_2} = 0.44$, $d_H = 0.0012$, $d_A = 0.153$, and $T_d = 59.94$ (2011- 31).
$\lambda_1 = 20000$, $\lambda_2 = 500$, $h_2 = 2500000$, $h_1 = 10000$, $c_1 = 0.0309$, $c_2 = 0.00012$, $r_H = 0.43$, $d_{L_1} = 0.34$, $d_{L_2} = 0.44$, $d_H = 0.0012$, $d_A = 0.153$, and $T_d = 59.94$ (2015- 45).
$\lambda_1 = 20000$, $\lambda_2 = 500$, $h_2 = 2500000$, $h_1 = 10000$, $c_1 = 0.0309$, $c_2 = 0.000212$, $r_H = 0.42$, $d_{L_1} = 0.34$, $d_{L_2} = 0.44$, $d_H = 0.0012$, $d_A = 0.153$, and $T_d = 59.94$ (2017- 78).
$\lambda_1 = 20000$, $\lambda_2 = 500$, $h_2 = 2500000$, $h_1 = 10000$, $c_1 = 0.0309$, $c_2 = 0.000204$, $r_H = 0.462$, $d_{L_1} = 0.34$, $d_{L_2} = 0.044$, $d_H = 0.0012$, $d_A = 0.153$, and $T_d = 59.94$ (2018- 134).

Figure 4(c) $\lambda_1 = 20000$, $\lambda_2 = 500$, $h_2 = 2500000$, $h_1 = 10000$, $c_1 = 0.0306$, $c_2 = 0.000096$, $r_H = 0.718$, $d_{L_1} = 0.33827$, $d_{L_2} = 0.48$, $d_H = 0.00138$, $d_A = 0.209$, and $T_d = 59.94$ (2013- 20).
$\lambda_1 = 20000$, $\lambda_2 = 500$, $h_2 = 2500000$, $h_1 = 10000$, $c_1 = 0.0309$, $c_2 = 0.0002$, $r_H = 0.55$, $d_{L_1} = 0.34$, $d_{L_2} = 0.44$, $d_H = 0.0012$, $d_A = 0.153$, and $T_d = 59.94$ (2016- 65).
$\lambda_1 = 20000$, $\lambda_2 = 500$, $h_2 = 2500000$, $h_1 = 10000$, $c_1 = 0.0309$, $c_2 = 0.0002$, $r_H = 0.5$, $d_{L_1} = 0.154$, $d_{L_2} = 0.344$, $d_H = 0.0012$, $d_A = 0.154$, and $T_d = 59.94$ (2018- 130).
$\lambda_1 = 20000$, $\lambda_2 = 500$, $h_2 = 2500000$, $h_1 = 10000$, $c_1 = 0.039$, $c_2 = 0.00022$, $r_H = 0.362$, $d_{L_1} = 0.154$, $d_{L_2} = 0.25$, $d_H = 0.0012$, $d_A = 0.154$, and $T_d = 59.94$ (2021- 200).

Figure 4(d) $\lambda_1 = 20000$, $\lambda_2 = 500$, $h_2 = 2500000$, $h_1 = 10000$, $c_1 = 0.0309$, $c_2 = 0.000097$, $r_H = 0.7$, $d_{L_1} = 0.247$, $d_{L_2} = 0.4$, $d_H = 0.0012$, $d_A = 0.193$, and $T_d = 60.94$ (2014- 33).
$\lambda_1 = 20000$, $\lambda_2 = 500$, $h_2 = 2500000$, $h_1 = 10000$, $c_1 = 0.0309$, $c_2 = 0.000097$, $r_H = 0.59$, $d_{L_1} = 0.234$, $d_{L_2} = 0.054$, $d_H = 0.0012$, $d_A = 0.153$, and $T_d = 60.94$ (2017- 66).
$\lambda_1 = 20000$, $\lambda_2 = 500$, $h_2 = 2500000$, $h_1 = 10000$, $c_1 = 0.0309$, $c_2 = 0.00016$, $r_H = 0.45$, $d_{L_1} = 0.15$, $d_{L_2} = 0.21$, $d_H = 0.0012$, $d_A = 0.153$, and $T_d = 59.94$ (2019- 129).
$\lambda_1 = 20000$, $\lambda_2 = 500$, $h_2 = 2500000$, $h_1 = 10000$, $c_1 = 0.0309$, $c_2 = 0.0002$, $r_H = 0.456$, $d_{L_1} = 0.148$, $d_{L_2} = 0.054$, $d_H = 0.0012$, $d_A = 0.153$, and $T_d = 60.94$ (2018- 218).

Figure 4(e) $\lambda_1 = 20000$, $\lambda_2 = 500$, $h_2 = 2500000$, $h_1 = 10000$, $c_1 = 0.0309$, $c_2 = 0.0000987$, $r_H = 0.64$, $d_{L_1} = 0.234$, $d_{L_2} = 0.294$, $d_H = 0.0012$, $d_A = 0.153$, and $T_d = 60.94$ (2016- 46).
$\lambda_1 = 20000$, $\lambda_2 = 500$, $h_2 = 2500000$, $h_1 = 10000$, $c_1 = 0.0309$, $c_2 = 0.0000987$, $r_H = 0.55$, $d_{L_1} = 0.234$, $d_{L_2} = 0.234$, $d_H = 0.0012$, $d_A = 0.153$, and $T_d = 60.94$ (2015- 61).
$\lambda_1 = 20000$, $\lambda_2 = 500$, $h_2 = 2500000$, $h_1 = 10000$, $c_1 = 0.0309$, $c_2 = 0.0002$, $r_H = 0.56$, $d_{L_1} = 0.234$, $d_{L_2} = 0.494$, $d_H = 0.0012$, $d_A = 0.153$, and $T_d = 60.94$ (2017- 98).
$\lambda_1 = 20000$, $\lambda_2 = 500$, $h_2 = 2500000$, $h_1 = 10000$, $c_1 = 0.0309$, $c_2 = 0.00029$, $r_H = 0.39$, $d_{L_1} = 0.1634$, $d_{L_2} = 0.094$, $d_H = 0.0012$, $d_A = 0.153$, and $T_d = 60.94$ (2018- 305).

Figure 4(f) $\lambda_1 = 20000$, $\lambda_2 = 500$, $h_2 = 2500000$, $h_1 = 10000$, $c_1 = 0.0309$, $c_2 = 0.0000987$, $r_H = 0.714$, $d_{L_1} = 0.3234$, $d_{L_2} = 0.4794$, $d_H = 0.0013$, $d_A = 0.193$, and $T_d = 60.94$ (2012- 25).
$\lambda_1 = 20000$, $\lambda_2 = 500$, $h_2 = 2500000$, $h_1 = 10000$, $c_1 = 0.0309$, $c_2 = 0.00016$, $r_H = 0.64$, $d_{L_1} = 0.234$, $d_{L_2} = 0.294$, $d_H = 0.0012$, $d_A = 0.153$, and $T_d = 60.94$ (2020- 73).
$\lambda_1 = 20000$, $\lambda_2 = 500$, $h_2 = 2500000$, $h_1 = 10000$, $c_1 = 0.0309$, $c_2 = 0.000223$, $r_H = 0.4$, $d_{L_1} = 0.234$, $d_{L_2} = 0.14$, $d_H = 0.0012$, $d_A = 0.153$, and $T_d = 60.94$ (2021- 174).
$\lambda_1 = 20000$, $\lambda_2 = 500$, $h_2 = 2500000$, $h_1 = 10000$, $c_1 = 0.0309$, $c_2 = 0.00029$, $r_H = 0.37$, $d_{L_1} = 0.234$, $d_{L_2} = 0.094$, $d_H = 0.0012$, $d_A = 0.153$, and $T_d = 60.94$ (2018- 261).

Figure 4(g) $\lambda_1 = 20000$, $\lambda_2 = 500$, $h_2 = 2500000$, $h_1 = 10000$, $c_1 = 0.0309$, $c_2 = 0.000127$, $r_H = 0.64$, $d_{L_1} = 0.234$, $d_{L_2} = 0.294$, $d_H = 0.0012$, $d_A = 0.153$, and $T_d = 60.94$ (2012- 58).
$\lambda_1 = 20000$, $\lambda_2 = 500$, $h_2 = 2500000$, $h_1 = 10000$, $c_1 = 0.0309$, $c_2 = 0.00017$, $r_H = 0.4$, $d_{L_1} = 0.234$, $d_{L_2} = 0.15$, $d_H = 0.0012$,

$d_A = 0.153$, and $T_d = 60.94$ (2020- 131).
$\lambda_1 = 20000$, $\lambda_2 = 500$, $h_2 = 2500000$, $h_1 = 10000$, $c_1 = 0.0309$, $c_2 = 0.000225$, $r_H = 0.41$, $d_{L_1} = 0.234$, $d_{L_2} = 0.094$, $d_H = 0.0012$, $d_A = 0.13$, and $T_d = 60.94$ (2017- 204).
$\lambda_1 = 20000$, $\lambda_2 = 500$, $h_2 = 2500000$, $h_1 = 10000$, $c_1 = 0.0309$, $c_2 = 0.000237$, $r_H = 0.4$, $d_{L_1} = 0.234$, $d_{L_2} = 0.1294$, $d_H = 0.0012$, $d_A = 0.0153$, and $T_d = 60.94$ (2016- 291).

Figure 4(h) $\lambda_1 = 20000$, $\lambda_2 = 500$, $h_2 = 2500000$, $h_1 = 10000$, $c_1 = 0.0309$, $c_2 = 0.0000987$, $r_H = 0.69$, $d_{L_1} = 0.234$, $d_{L_2} = 0.34$, $d_H = 0.0012$, $d_A = 0.153$, and $T_d = 60.94$ (2019- 41).
$\lambda_1 = 20000$, $\lambda_2 = 500$, $h_2 = 2500000$, $h_1 = 10000$, $c_1 = 0.0309$, $c_2 = 0.00017$, $r_H = 0.64$, $d_{L_1} = 0.234$, $d_{L_2} = 0.294$, $d_H = 0.0012$, $d_A = 0.153$, and $T_d = 60.94$ (2018- 77).
$\lambda_1 = 20000$, $\lambda_2 = 500$, $h_2 = 2500000$, $h_1 = 10000$, $c_1 = 0.0309$, $c_2 = 0.0002$, $r_H = 0.51$, $d_{L_1} = 0.234$, $d_{L_2} = 0.094$, $d_H = 0.0012$, $d_A = 0.1153$, and $T_d = 60.94$ (2012- 158).
$\lambda_1 = 20000$, $\lambda_2 = 500$, $h_2 = 2500000$, $h_1 = 10000$, $c_1 = 0.0309$, $c_2 = 0.0002$, $r_H = 0.41$, $d_{L_1} = 0.234$, $d_{L_2} = 0.094$, $d_H = 0.0012$, $d_A = 0.053$, and $T_d = 60.94$ (2021- 231).

Figure 4(i) $\lambda_1 = 20000$, $\lambda_2 = 500$, $h_2 = 2500000$, $h_1 = 10000$, $c_1 = 0.0309$, $c_2 = 0.000296$, $r_H = 0.4$, $d_{L_1} = 0.234$, $d_{L_2} = 0.294$, $d_H = 0.0012$, $d_A = 0.153$, and $T_d = 60.94$ (2011- 170).
$\lambda_1 = 20000$, $\lambda_2 = 500$, $h_2 = 2500000$, $h_1 = 10000$, $c_1 = 0.0309$, $c_2 = 0.00036$, $r_H = 0.4$, $d_{L_1} = 0.234$, $d_{L_2} = 0.13$, $d_H = 0.0012$, $d_A = 0.1153$, and $T_d = 60.94$ (2013- 309).
$\lambda_1 = 20000$, $\lambda_2 = 500$, $h_2 = 2500000$, $h_1 = 10000$, $c_1 = 0.0409$, $c_2 = 0.00033$, $r_H = 0.4$, $d_{L_1} = 0.234$, $d_{L_2} = 0.094$, $d_H = 0.0012$, $d_A = 0.0153$, and $T_d = 60.94$ (2017- 430).
$\lambda_1 = 20000$, $\lambda_2 = 500$, $h_2 = 2500000$, $h_1 = 10000$, $c_1 = 0.0419$, $c_2 = 0.00035$, $r_H = 0.36$, $d_{L_1} = 0.16$, $d_{L_2} = 0.064$, $d_H = 0.0012$, $d_A = 0.0153$, and $T_d = 60.94$ (2016- 750).

Figure 6(a) $\lambda_1 = 20000$, $\lambda_2 = 600$, $h_2 = 2500000$, $h_1 = 10000$, $c_1 = 0.0309$, $c_2 = 0.000098$, $r_H = 0.64$, $d_{L_1} = 0.33$, $d_{L_2} = 0.25$, $d_H = 0.0012$, $d_A = 0.19$, and $T_d = 59.94$ (2012- 158).
$\lambda_1 = 20000$, $\lambda_2 = 6000$, $h_2 = 2500000$, $h_1 = 10000$, $c_1 = 0.0309$, $c_2 = 0.000098$, $r_H = 0.66$, $d_{L_1} = 0.33$, $d_{L_2} = 0.04$, $d_H = 0.0012$, $d_A = 0.19$, and $T_d = 59.94$ (2021- 210).
$\lambda_1 = 20000$, $\lambda_2 = 6000$, $h_2 = 2500000$, $h_1 = 10000$, $c_1 = 0.0309$, $c_2 = 0.000305$, $r_H = 0.46$, $d_{L_1} = 0.33$, $d_{L_2} = 0.04$, $d_H = 0.0012$, $d_A = 0.19$, and $T_d = 59.94$ (2017- 900).
$\lambda_1 = 20000$, $\lambda_2 = 6000$, $h_2 = 2500000$, $h_1 = 10000$, $c_1 = 0.0309$, $c_2 = 0.000336$, $r_H = 0.46$, $d_{L_1} = 0.33$, $d_{L_2} = 0.04$, $d_H = 0.0012$, $d_A = 0.19$, and $T_d = 59.94$ (2020- 990).

Figure 6(b) $\lambda_1 = 20000$, $\lambda_2 = 6000$, $h_2 = 2500000$, $h_1 = 10000$, $c_1 = 0.0309$, $c_2 = 0.00012$, $r_H = 0.64$, $d_{L_1} = 0.33$, $d_{L_2} = 0.25$, $d_H = 0.0012$, $d_A = 0.19$, and $T_d = 59.94$ (2014- 200).
$\lambda_1 = 20000$, $\lambda_2 = 6000$, $h_2 = 2500000$, $h_1 = 10000$, $c_1 = 0.0309$, $c_2 = 0.00014$, $r_H = 0.59$, $d_{L_1} = 0.33$, $d_{L_2} = 0.25$, $d_H = 0.0012$, $d_A = 0.19$, and $T_d = 59.94$ (2015- 245).
$\lambda_1 = 20000$, $\lambda_2 = 6000$, $h_2 = 2500000$, $h_1 = 10000$, $c_1 = 0.0309$, $c_2 = 0.0003$, $r_H = 0.41$, $d_{L_1} = 0.33$, $d_{L_2} = 0.05$, $d_H = 0.0012$, $d_A = 0.19$, and $T_d = 59.94$ (2011- 920).
$\lambda_1 = 20000$, $\lambda_2 = 6000$, $h_2 = 2500000$, $h_1 = 10000$, $c_1 = 0.0309$, $c_2 = 0.0003$, $r_H = 0.43$, $d_{L_1} = 0.33$, $d_{L_2} = 0.05$, $d_H = 0.0012$, $d_A = 0.19$, and $T_d = 59.94$ (2013- 960).

Figure 6(c) $\lambda_1 = 20000$, $\lambda_2 = 6000$, $h_2 = 2500000$, $h_1 = 10000$, $c_1 = 0.0309$, $c_2 = 0.00012$, $r_H = 0.4$, $d_{L_1} = 0.33$, $d_{L_2} = 0.25$, $d_H = 0.0012$, $d_A = 0.19$, and $T_d = 59.94$ (2016- 230).
$\lambda_1 = 20000$, $\lambda_2 = 6000$, $h_2 = 2500000$, $h_1 = 10000$, $c_1 = 0.0309$, $c_2 = 0.000208$, $r_H = 0.64$, $d_{L_1} = 0.33$, $d_{L_2} = 0.25$, $d_H = 0.0012$, $d_A = 0.19$, and $T_d = 59.94$ (2018- 340).
$\lambda_1 = 20000$, $\lambda_2 = 500$, $h_2 = 2500000$, $h_1 = 10000$, $c_1 = 0.0309$, $c_2 = 0.000186$, $r_H = 0.4$, $d_{L_1} = 0.33$, $d_{L_2} = 0.05$, $d_H = 0.0012$, $d_A = 0.019$, and $T_d = 59.94$ (2019- 940).

Figure 6(e) $\lambda_1 = 20000$, $\lambda_2 = 6000$, $h_2 = 2500000$, $h_1 = 10000$, $c_1 = 0.0309$, $c_2 = 0.0000987$, $r_H = 0.62$, $d_{L_1} = 0.33$, $d_{L_2} = 0.04$, $d_H = 0.0012$, $d_A = 0.19$, and $T_d = 59.94$ (2013- 220).
$\lambda_1 = 20000$, $\lambda_2 = 6000$, $h_2 = 2500000$, $h_1 = 10000$, $c_1 = 0.0309$, $c_2 = 0.00012$, $r_H = 0.46$, $d_{L_1} = 0.33$, $d_{L_2} = 0.04$, $d_H = 0.0012$, $d_A = 0.19$, and $T_d = 59.94$ (2021- 355).
$\lambda_1 = 20000$, $\lambda_2 = 6000$, $h_2 = 2500000$, $h_1 = 10000$, $c_1 = 0.0309$, $c_2 = 0.000241$, $r_H = 0.46$, $d_{L_1} = 0.33$, $d_{L_2} = 0.04$, $d_H = 0.0012$, $d_A = 0.19$, and $T_d = 59.94$ (2016- 713).

$\lambda_1 = 20000$, $\lambda_2 = 6000$, $h_2 = 2500000$, $h_1 = 10000$, $c_1 = 0.0309$, $c_2 = 0.000315$, $r_H = 0.46$, $d_{L_1} = 0.33$, $d_{L_2} = 0.04$, $d_H = 0.0012$, $d_A = 0.19$, and $T_d = 59.94$ (2017- 926).

Figure 6(f) $\lambda_1 = 20000$, $\lambda_2 = 6000$, $h_2 = 2500000$, $h_1 = 10000$, $c_1 = 0.0309$, $c_2 = 0.000098$, $r_H = 0.6$, $d_{L_1} = 0.33$, $d_{L_2} = 0.04$, $d_H = 0.0012$, $d_A = 0.19$, and $T_d = 59.94$ (2011-224).
$\lambda_1 = 20000$, $\lambda_2 = 6000$, $h_2 = 2500000$, $h_1 = 10000$, $c_1 = 0.0309$, $c_2 = 0.000108$, $r_H = 0.46$, $d_{L_1} = 0.33$, $d_{L_2} = 0.04$, $d_H = 0.0012$, $d_A = 0.19$, and $T_d = 59.94$ (2020- 318).
$\lambda_1 = 20000$, $\lambda_2 = 6000$, $h_2 = 2500000$, $h_1 = 10000$, $c_1 = 0.0309$, $c_2 = 0.000127$, $r_H = 0.46$, $d_{L_1} = 0.33$, $d_{L_2} = 0.04$, $d_H = 0.0012$, $d_A = 0.19$, and $T_d = 59.94$ (2015- 375).
$\lambda_1 = 20000$, $\lambda_2 = 6000$, $h_2 = 2500000$, $h_1 = 10000$, $c_1 = 0.0309$, $c_2 = 0.000261$, $r_H = 0.46$, $d_{L_1} = 0.33$, $d_{L_2} = 0.04$, $d_H = 0.0012$, $d_A = 0.19$, and $T_d = 59.94$ (2018- 773).

Figure 6(g) $\lambda_1 = 20000$, $\lambda_2 = 6000$, $h_2 = 2500000$, $h_1 = 10000$, $c_1 = 0.0309$, $c_2 = 0.0001024$, $r_H = 0.46$, $d_{L_1} = 0.33$, $d_{L_2} = 0.04$, $d_H = 0.0012$, $d_A = 0.19$, and $T_d = 59.94$ (2014- 300).
$\lambda_1 = 20000$, $\lambda_2 = 6000$, $h_2 = 2500000$, $h_1 = 10000$, $c_1 = 0.0309$, $c_2 = 0.000116$, $r_H = 0.46$, $d_{L_1} = 0.33$, $d_{L_2} = 0.04$, $d_H = 0.0012$, $d_A = 0.19$, and $T_d = 59.94$ (2019- 345).
$\lambda_1 = 20000$, $\lambda_2 = 6000$, $h_2 = 2500000$, $h_1 = 10000$, $c_1 = 0.0309$, $c_2 = 0.000166$, $r_H = 0.46$, $d_{L_1} = 0.33$, $d_{L_2} = 0.04$, $d_H = 0.0012$, $d_A = 0.19$, and $T_d = 59.94$ (2012- 490).

Figure 6(i) $\lambda_1 = 20000$, $\lambda_2 = 6000$, $h_2 = 2500000$, $h_1 = 10000$, $c_1 = 0.0309$, $c_2 = 0.000109$, $r_H = 0.5$, $d_{L_1} = 0.153$, $d_{L_2} = 0.04$, $d_H = 0.0012$, $d_A = 0.05$, and $T_d = 60.94$ (2015- 800).
$\lambda_1 = 20000$, $\lambda_2 = 6000$, $h_2 = 2500000$, $h_1 = 10000$, $c_1 = 0.0309$, $c_2 = 0.0001$, $r_H = 0.35$, $d_{L_1} = 0.153$, $d_{L_2} = 0.04$, $d_H = 0.0012$, $d_A = 0.05$, and $T_d = 60.94$ (2018- 1036).
$\lambda_1 = 20000$, $\lambda_2 = 6000$, $h_2 = 2500000$, $h_1 = 10000$, $c_1 = 0.0309$, $c_2 = 0.000238$, $r_H = 0.35$, $d_{L_1} = 0.153$, $d_{L_2} = 0.04$, $d_H = 0.0012$, $d_A = 0.019$, and $T_d = 60.94$ (2017- 2833).
$\lambda_1 = 20000$, $\lambda_2 = 6000$, $h_2 = 2500000$, $h_1 = 10000$, $c_1 = 0.0309$, $c_2 = 0.000284$, $r_H = 0.35$, $d_{L_1} = 0.153$, $d_{L_2} = 0.04$, $d_H = 0.0012$, $d_A = 0.019$, and $T_d = 60.94$ (2016- 3410).

Figure 6(j) $\lambda_1 = 20000$, $\lambda_2 = 6000$, $h_2 = 2500000$, $h_1 = 10000$, $c_1 = 0.0309$, $c_2 = 0.000098$, $r_H = 0.58$, $d_{L_1} = 0.153$, $d_{L_2} = 0.34$, $d_H = 0.0012$, $d_A = 0.15$, and $T_d = 60.94$ (2014- 278).
$\lambda_1 = 20000$, $\lambda_2 = 6000$, $h_2 = 2500000$, $h_1 = 10000$, $c_1 = 0.0309$, $c_2 = 0.000109$, $r_H = 0.55$, $d_{L_1} = 0.153$, $d_{L_2} = 0.04$, $d_H = 0.0012$, $d_A = 0.15$, and $T_d = 60.94$ (2013- 487).
$\lambda_1 = 20000$, $\lambda_2 = 6000$, $h_2 = 2500000$, $h_1 = 10000$, $c_1 = 0.0309$, $c_2 = 0.0001$, $r_H = 0.54$, $d_{L_1} = 0.153$, $d_{L_2} = 0.04$, $d_H = 0.0012$, $d_A = 0.05$, and $T_d = 60.94$ (2012- 680).
$\lambda_1 = 20000$, $\lambda_2 = 6000$, $h_2 = 2500000$, $h_1 = 10000$, $c_1 = 0.0309$, $c_2 = 0.000107$, $r_H = 0.5$, $d_{L_1} = 0.153$, $d_{L_2} = 0.04$, $d_H = 0.0012$, $d_A = 0.05$, and $T_d = 60.94$ (2011- 790).

Figure 6(k) $\lambda_1 = 20000$, $\lambda_2 = 6000$, $h_2 = 2500000$, $h_1 = 10000$, $c_1 = 0.0309$, $c_2 = 0.0003$, $r_H = 0.58$, $d_{L_1} = 0.153$, $d_{L_2} = 0.34$, $d_H = 0.0012$, $d_A = 0.15$, and $T_d = 60.94$ (2021- 850).
$\lambda_1 = 20000$, $\lambda_2 = 6000$, $h_2 = 2500000$, $h_1 = 10000$, $c_1 = 0.0309$, $c_2 = 0.00035$, $r_H = 0.58$, $d_{L_1} = 0.153$, $d_{L_2} = 0.34$, $d_H = 0.0012$, $d_A = 0.15$, and $T_d = 60.94$ (2019- 990).
$\lambda_1 = 20000$, $\lambda_2 = 6000$, $h_2 = 2500000$, $h_1 = 10000$, $c_1 = 0.0409$, $c_2 = 0.000365$, $r_H = 0.45$, $d_{L_1} = 0.153$, $d_{L_2} = 0.34$, $d_H = 0.0012$, $d_A = 0.15$, and $T_d = 60.94$ (2020- 1200).

Figure 6(m) $\lambda_1 = 20000$, $\lambda_2 = 6000$, $h_2 = 2500000$, $h_1 = 10000$, $c_1 = 0.0309$, $c_2 = 0.000284$, $r_H = 0.6$, $d_{L_1} = 0.33$, $d_{L_2} = 0.47$, $d_H = 0.0012$, $d_A = 0.19$, and $T_d = 60.94$ (2011- 40).
$\lambda_1 = 20000$, $\lambda_2 = 6000$, $h_2 = 2500000$, $h_1 = 10000$, $c_1 = 0.0309$, $c_2 = 0.0000987$, $r_H = 0.68$, $d_{L_1} = 0.33$, $d_{L_2} = 0.4$, $d_H = 0.0012$, $d_A = 0.19$, and $T_d = 60.94$ (2013- 136).
$\lambda_1 = 20000$, $\lambda_2 = 6000$, $h_2 = 2500000$, $h_1 = 10000$, $c_1 = 0.0309$, $c_2 = 0.0001$, $r_H = 0.68$, $d_{L_1} = 0.34$, $d_{L_2} = 0.34$, $d_H = 0.0012$, $d_A = 0.15$, and $T_d = 60.94$ (2020- 200).
$\lambda_1 = 20000$, $\lambda_2 = 6000$, $h_2 = 2500000$, $h_1 = 10000$, $c_1 = 0.0309$, $c_2 = 0.000098$, $r_H = 0.63$, $d_{L_1} = 0.153$, $d_{L_2} = 0.34$, $d_H = 0.0012$, $d_A = 0.15$, and $T_d = 60.94$ (2019- 266).

Figure 6(n) $\lambda_1 = 20000$, $\lambda_2 = 6000$, $h_2 = 2500000$, $h_1 = 10000$, $c_1 = 0.0309$, $c_2 = 0.000197$, $r_H = 0.68$, $d_{L_1} = 0.33$, $d_{L_2} = 0.4$, $d_H = 0.0012$, $d_A = 0.19$, and $T_d = 60.94$ (2021- 28).

$\lambda_1 = 20000$, $\lambda_2 = 6000$, $h_2 = 2500000$, $h_1 = 10000$, $c_1 = 0.0309$, $c_2 = 0.000498$, $r_H = 0.68$, $d_{L_1} = 0.33$, $d_{L_2} = 0.478$, $d_H = 0.0012$, $d_A = 0.19$, and $T_d = 60.94$ (2015- 64).
$\lambda_1 = 20000$, $\lambda_2 = 6000$, $h_2 = 2500000$, $h_1 = 10000$, $c_1 = 0.0309$, $c_2 = 0.000143$, $r_H = 0.68$, $d_{L_1} = 0.33$, $d_{L_2} = 0.4$, $d_H = 0.0012$, $d_A = 0.19$, and $T_d = 60.94$ (2016- 199).
$\lambda_1 = 20000$, $\lambda_2 = 6000$, $h_2 = 2500000$, $h_1 = 10000$, $c_1 = 0.0309$, $c_2 = 0.00017$, $r_H = 0.68$, $d_{L_1} = 0.33$, $d_{L_2} = 0.4$, $d_H = 0.0012$, $d_A = 0.14$, and $T_d = 60.94$ (2017- 266).

Figure 6(o) $\lambda_1 = 20000$, $\lambda_2 = 6000$, $h_2 = 2500000$, $h_1 = 10000$, $c_1 = 0.0309$, $c_2 = 0.000038$, $r_H = 0.7$, $d_{L_1} = 0.33$, $d_{L_2} = 0.43$, $d_H = 0.0012$, $d_A = 0.19$, and $T_d = 60.94$ (2014- 50).
$\lambda_1 = 20000$, $\lambda_2 = 6000$, $h_2 = 2500000$, $h_1 = 10000$, $c_1 = 0.0309$, $c_2 = 0.000161$, $r_H = 0.68$, $d_{L_1} = 0.33$, $d_{L_2} = 0.4$, $d_H = 0.0012$, $d_A = 0.19$, and $T_d = 60.94$ (2012- 224).
$\lambda_1 = 20000$, $\lambda_2 = 6000$, $h_2 = 2500000$, $h_1 = 10000$, $c_1 = 0.0309$, $c_2 = 0.000187$, $r_H = 0.68$, $d_{L_1} = 0.33$, $d_{L_2} = 0.4$, $d_H = 0.0012$, $d_A = 0.19$, and $T_d = 60.94$ (2018- 256).

Figure S5(a) $\lambda_1 = 20000$, $\lambda_2 = 500$, $h_2 = 2500000$, $h_1 = 10000$, $c_1 = 0.0309$, $c_2 = 0.0001$, $r_H = 0.53$, $d_{L_1} = 0.189$, $d_{L_2} = 0.4$, $d_H = 0.0012$, $d_A = 0.13$, and $T_d = 59.94$ (January- 55).

Figure S5(b) $\lambda_1 = 20000$, $\lambda_2 = 500$, $h_2 = 2500000$, $h_1 = 10000$, $c_1 = 0.0309$, $c_2 = 0.000097$, $r_H = 0.6$, $d_{L_1} = 0.33$, $d_{L_2} = 0.4$, $d_H = 0.0012$, $d_A = 0.19$, and $T_d = 59.94$ (February- 30).

Figure S5(c) $\lambda_1 = 20000$, $\lambda_2 = 500$, $h_2 = 2500000$, $h_1 = 10000$, $c_1 = 0.0309$, $c_2 = 0.000097$, $r_H = 0.6$, $d_{L_1} = 0.33$, $d_{L_2} = 0.4$, $d_H = 0.0012$, $d_A = 0.19$, and $T_d = 59.94$ (March- 30).

Figure S5(d) $\lambda_1 = 20000$, $\lambda_2 = 500$, $h_2 = 2500000$, $h_1 = 10000$, $c_1 = 0.0309$, $c_2 = 0.0000964$, $r_H = 0.71$, $d_{L_1} = 0.338$, $d_{L_2} = 0.46$, $d_H = 0.0012$, $d_A = 0.19$, and $T_d = 59.94$ (April- 25).

Figure S5(e) $\lambda_1 = 20000$, $\lambda_2 = 500$, $h_2 = 2500000$, $h_1 = 10000$, $c_1 = 0.04109$, $c_2 = 0.000098$, $r_H = 0.58$, $d_{L_1} = 0.3$, $d_{L_2} = 0.24$, $d_H = 0.0012$, $d_A = 0.19$, and $T_d = 59.94$ (May- 40).

Figure S5(f) $\lambda_1 = 20000$, $\lambda_2 = 500$, $h_2 = 2500000$, $h_1 = 10000$, $c_1 = 0.0309$, $c_2 = 0.00013$, $r_H = 0.4$, $d_{L_1} = 0.33$, $d_{L_2} = 0.4$, $d_H = 0.0012$, $d_A = 0.019$, and $T_d = 59.94$ (June- 75).

Figure S5(g) $\lambda_1 = 20000$, $\lambda_2 = 500$, $h_2 = 2500000$, $h_1 = 10000$, $c_1 = 0.0309$, $c_2 = 0.00019$, $r_H = 0.6$, $d_{L_1} = 0.27$, $d_{L_2} = 0.4$, $d_H = 0.0012$, $d_A = 0.19$, and $T_d = 59.94$ (July- 65).

Figure S5(h) $\lambda_1 = 20000$, $\lambda_2 = 500$, $h_2 = 2500000$, $h_1 = 10000$, $c_1 = 0.0309$, $c_2 = 0.0002$, $r_H = 0.35$, $d_{L_1} = 0.27$, $d_{L_2} = 0.4$, $d_H = 0.0012$, $d_A = 0.1243$, and $T_d = 59.94$ (August- 105).

Figure S5(i) $\lambda_1 = 20000$, $\lambda_2 = 500$, $h_2 = 2500000$, $h_1 = 10000$, $c_1 = 0.0309$, $c_2 = 0.00019$, $r_H = 0.36$, $d_{L_1} = 0.27$, $d_{L_2} = 0.24$, $d_H = 0.0012$, $d_A = 0.026$, and $T_d = 59.94$ (September- 170).

Figure S5(j) $\lambda_1 = 20000$, $\lambda_2 = 500$, $h_2 = 2500000$, $h_1 = 10000$, $c_1 = 0.0309$, $c_2 = 0.0002$, $r_H = 0.438$, $d_{L_1} = 0.27$, $d_{L_2} = 0.24$, $d_H = 0.0012$, $d_A = 0.019$, and $T_d = 60.94$ (October- 165).

Figure S5(k) $\lambda_1 = 20000$, $\lambda_2 = 500$, $h_2 = 2500000$, $h_1 = 10000$, $c_1 = 0.0309$, $c_2 = 0.00019$, $r_H = 0.51$, $d_{L_1} = 0.27$, $d_{L_2} = 0.24$, $d_H = 0.0012$, $d_A = 0.1$, and $T_d = 59.94$ (November- 110).

Figure S5(l) $\lambda_1 = 20000$, $\lambda_2 = 500$, $h_2 = 2500000$, $h_1 = 10000$, $c_1 = 0.0409$, $c_2 = 0.000103$, $r_H = 0.399$, $d_{L_1} = 0.27$, $d_{L_2} = 0.24$, $d_H = 0.0012$, $d_A = 0.11$, and $T_d = 59.94$ (December- 70).

Figure S6(a) $\lambda_1 = 20000$, $\lambda_2 = 500$, $h_2 = 2500000$, $h_1 = 10000$, $c_1 = 0.039$, $c_2 = 0.0001$, $r_H = 0.6$, $d_{L_1} = 0.29$, $d_{L_2} = 0.043$, $d_H = 0.0012$, $d_A = 0.18$, and $T_d = 59.94$ (January- 55).

Figure S6(b) $\lambda_1 = 20000$, $\lambda_2 = 500$, $h_2 = 2500000$, $h_1 = 10000$, $c_1 = 0.039$, $c_2 = 0.000098$, $r_H = 0.7$, $d_{L_1} = 0.29$, $d_{L_2} = 0.43$, $d_H = 0.0012$, $d_A = 0.18$, and $T_d = 59.94$ (February- 30).

Figure S6(c) $\lambda_1 = 20000$, $\lambda_2 = 500$, $h_2 = 2500000$, $h_1 = 10000$, $c_1 = 0.039$, $c_2 = 0.000134$, $r_H = 0.4$, $d_{L_1} = 0.29$, $d_{L_2} = 0.43$, $d_H = 0.0012$, $d_A = 0.18$, and $T_d = 59.94$ (March- 55).

Figure S6(d) $\lambda_1 = 20000$, $\lambda_2 = 500$, $h_2 = 2500000$, $h_1 = 10000$, $c_1 = 0.039$, $c_2 = 0.0000965$, $r_H = 0.71$, $d_{L_1} = 0.3289$, $d_{L_2} = 0.472$, $d_H = 0.0012$, $d_A = 0.198$, and $T_d = 59.94$ (April- 25).

Figure S6(e) $\lambda_1 = 20000$, $\lambda_2 = 500$, $h_2 = 2500000$, $h_1 = 10000$, $c_1 = 0.039$, $c_2 = 0.000098$, $r_H = 0.7$, $d_{L_1} = 0.29$, $d_{L_2} = 0.43$, $d_H = 0.0012$, $d_A = 0.18$, and $T_d = 59.94$ (May- 30).

Figure S6(f) $\lambda_1 = 20000$, $\lambda_2 = 500$, $h_2 = 2500000$, $h_1 = 10000$, $c_1 = 0.039$, $c_2 = 0.000098$, $r_H = 0.4$, $d_{L_1} = 0.29$, $d_{L_2} = 0.283$, $d_H = 0.0012$, $d_A = 0.18$, and $T_d = 59.94$ (June- 50).

Figure S6(g) $\lambda_1 = 20000$, $\lambda_2 = 500$, $h_2 = 2500000$, $h_1 = 10000$, $c_1 = 0.039$, $c_2 = 0.00011$, $r_H = 0.4$, $d_{L_1} = 0.19$, $d_{L_2} = 0.143$, $d_H = 0.0012$, $d_A = 0.18$, and $T_d = 59.94$ (July- 90).

Figure S6(h) $\lambda_1 = 20000$, $\lambda_2 = 500$, $h_2 = 2500000$, $h_1 = 10000$, $c_1 = 0.039$, $c_2 = 0.0001$, $r_H = 0.62$, $d_{L_1} = 0.189$, $d_{L_2} = 0.04$, $d_H = 0.0012$, $d_A = 0.13$, and $T_d = 59.94$ (August- 80).

Figure S6(i) $\lambda_1 = 20000$, $\lambda_2 = 500$, $h_2 = 2500000$, $h_1 = 10000$, $c_1 = 0.039$, $c_2 = 0.00012$, $r_H = 0.4$, $d_{L_1} = 0.29$, $d_{L_2} = 0.143$, $d_H = 0.0012$, $d_A = 0.18$, and $T_d = 59.94$ (September- 80).

Figure S6(j) $\lambda_1 = 20000$, $\lambda_2 = 500$, $h_2 = 2500000$, $h_1 = 10000$, $c_1 = 0.039$, $c_2 = 0.00012$, $r_H = 0.4$, $d_{L_1} = 0.19$, $d_{L_2} = 0.088$, $d_H = 0.0012$, $d_A = 0.18$, and $T_d = 59.94$ (October- 90).

Figure S6(k) $\lambda_1 = 20000$, $\lambda_2 = 500$, $h_2 = 2500000$, $h_1 = 10000$, $c_1 = 0.039$, $c_2 = 0.000098$, $r_H = 0.4$, $d_{L_1} = 0.19$, $d_{L_2} = 0.143$, $d_H = 0.0012$, $d_A = 0.18$, and $T_d = 59.94$ (November- 80).

Figure S6(l) $\lambda_1 = 20000$, $\lambda_2 = 500$, $h_2 = 2500000$, $h_1 = 10000$, $c_1 = 0.039$, $c_2 = 0.0001$, $r_H = 0.62$, $d_{L_1} = 0.189$, $d_{L_2} = 0.04$, $d_H = 0.0012$, $d_A = 0.189$, and $T_d = 59.94$ (December- 70).

Figure S7(a) $\lambda_1 = 20000$, $\lambda_2 = 500$, $h_2 = 2500000$, $h_1 = 10000$, $c_1 = 0.0309$, $c_2 = 0.0000964$, $r_H = 0.7$, $d_{L_1} = 0.33$, $d_{L_2} = 0.46$, $d_H = 0.0012$, $d_A = 0.199$, and $T_d = 59.98$ (January- 25).

Figure S7(b) $\lambda_1 = 20000$, $\lambda_2 = 500$, $h_2 = 2500000$, $h_1 = 10000$, $c_1 = 0.0309$, $c_2 = 0.0000964$, $r_H = 0.6$, $d_{L_1} = 0.33$, $d_{L_2} = 0.4$, $d_H = 0.0012$, $d_A = 0.19$, and $T_d = 59.98$ (February- 30).

Figure S7(c) $\lambda_1 = 20000$, $\lambda_2 = 500$, $h_2 = 2500000$, $h_1 = 10000$, $c_1 = 0.0309$, $c_2 = 0.000098$, $r_H = 0.4109$, $d_{L_1} = 0.33$, $d_{L_2} = 0.26$, $d_H = 0.0012$, $d_A = 0.19$, and $T_d = 59.98$ (March- 45).

Figure S7(d) $\lambda_1 = 20000$, $\lambda_2 = 500$, $h_2 = 2500000$, $h_1 = 10000$, $c_1 = 0.0309$, $c_2 = 0.00021$, $r_H = 0.49$, $d_{L_1} = 0.33$, $d_{L_2} = 0.24$, $d_H = 0.0012$, $d_A = 0.19$, and $T_d = 60.94$ (April- 90).

Figure S7(e) $\lambda_1 = 20000$, $\lambda_2 = 500$, $h_2 = 2500000$, $h_1 = 10000$, $c_1 = 0.0309$, $c_2 = 0.000184$, $r_H = 0.4$, $d_{L_1} = 0.33$, $d_{L_2} = 0.26$, $d_H = 0.0012$, $d_A = 0.19$, and $T_d = 59.98$ (May- 85).

Figure S7(f) $\lambda_1 = 20000$, $\lambda_2 = 500$, $h_2 = 2500000$, $h_1 = 10000$, $c_1 = 0.0309$, $c_2 = 0.000173$, $r_H = 0.4$, $d_{L_1} = 0.33$, $d_{L_2} = 0.26$, $d_H = 0.0012$, $d_A = 0.19$, and $T_d = 59.98$ (June- 80).

Figure S7(g) $\lambda_1 = 20000$, $\lambda_2 = 500$, $h_2 = 2500000$, $h_1 = 10000$, $c_1 = 0.0309$, $c_2 = 0.00023$, $r_H = 0.4$, $d_{L_1} = 0.33$, $d_{L_2} = 0.26$, $d_H = 0.0012$, $d_A = 0.19$, and $T_d = 59.98$ (July- 105).

Figure S7(h) $\lambda_1 = 20000$, $\lambda_2 = 500$, $h_2 = 2500000$, $h_1 = 10000$, $c_1 = 0.0309$, $c_2 = 0.00021$, $r_H = 0.486$, $d_{L_1} = 0.33$, $d_{L_2} = 0.14$, $d_H = 0.0012$, $d_A = 0.19$, and $T_d = 60.94$ (August- 105).

Figure S7(i) $\lambda_1 = 20000$, $\lambda_2 = 500$, $h_2 = 2500000$, $h_1 = 10000$, $c_1 = 0.0309$, $c_2 = 0.00013$, $r_H = 0.4$, $d_{L_1} = 0.33$, $d_{L_2} = 0.26$, $d_H = 0.0013$, $d_A = 0.19$, and $T_d = 59.98$ (September- 60).

Figure S7(j) $\lambda_1 = 20000$, $\lambda_2 = 500$, $h_2 = 2500000$, $h_1 = 10000$, $c_1 = 0.0309$, $c_2 = 0.000162$, $r_H = 0.4$, $d_{L_1} = 0.33$, $d_{L_2} = 0.26$, $d_H = 0.0012$, $d_A = 0.19$, and $T_d = 59.98$ (October- 75).

Figure S7(k) $\lambda_1 = 20000$, $\lambda_2 = 500$, $h_2 = 2500000$, $h_1 = 10000$, $c_1 = 0.0309$, $c_2 = 0.000118$, $r_H = 0.4$, $d_{L_1} = 0.33$, $d_{L_2} = 0.26$, $d_H = 0.0012$, $d_A = 0.19$, and $T_d = 59.98$ (November- 55).

Figure S7(l) $\lambda_1 = 20000$, $\lambda_2 = 500$, $h_2 = 2500000$, $h_1 = 10000$, $c_1 = 0.0309$, $c_2 = 0.00014$, $r_H = 0.49$, $d_{L_1} = 0.33$, $d_{L_2} = 0.24$, $d_H = 0.0012$, $d_A = 0.19$, and $T_d = 60.94$ (December- 60).

Figure S8(a)$\lambda_1 = 20000$, $\lambda_2 = 500$, $h_2 = 2500000$, $h_1 = 10000$, $c_1 = 0.0309$, $c_2 = 0.000098$, $r_H = 0.7$, $d_{L_1} = 0.33$, $d_{L_2} = 0.32$, $d_H = 0.0012$, $d_A = 0.19$, and $T_d = 59.98$ (January- 30).

Figure S8(b) $\lambda_1 = 20000$, $\lambda_2 = 500$, $h_2 = 2500000$, $h_1 = 10000$, $c_1 = 0.0309$, $c_2 = 0.00009634$, $r_H = 0.718$, $d_{L_1} = 0.3379$, $d_{L_2} = 0.48$, $d_H = 0.0012$, $d_A = 0.209$, and $T_d = 59.98$ (February- 20).

Figure S8(c) $\lambda_1 = 20000$, $\lambda_2 = 500$, $h_2 = 2500000$, $h_1 = 10000$, $c_1 = 0.0309$, $c_2 = 0.000106$, $r_H = 0.4$, $d_{L_1} = 0.33$, $d_{L_2} = 0.26$, $d_H = 0.0012$, $d_A = 0.19$, and $T_d = 59.98$ (March- 50).

Figure S8(d) $\lambda_1 = 20000$, $\lambda_2 = 500$, $h_2 = 2500000$, $h_1 = 10000$, $c_1 = 0.0309$, $c_2 = 0.00009813$, $r_H = 0.61$, $d_{L_1} = 0.33$, $d_{L_2} = 0.26$, $d_H = 0.0012$, $d_A = 0.19$, and $T_d = 59.98$ (April- 35).

Figure S8(e) $\lambda_1 = 20000$, $\lambda_2 = 500$, $h_2 = 2500000$, $h_1 = 10000$, $c_1 = 0.0309$, $c_2 = 0.000173$, $r_H = 0.4$, $d_{L_1} = 0.33$, $d_{L_2} = 0.26$, $d_H = 0.0012$, $d_A = 0.19$, and $T_d = 59.98$ (May- 80).

Figure S8(f) $\lambda_1 = 20000$, $\lambda_2 = 500$, $h_2 = 2500000$, $h_1 = 10000$, $c_1 = 0.0309$, $c_2 = 0.00016$, $r_H = 0.4$, $d_{L_1} = 0.33$, $d_{L_2} = 0.06$, $d_H = 0.0012$, $d_A = 0.129$, and $T_d = 59.98$ (June- 125).

Figure S8(g) $\lambda_1 = 20000$, $\lambda_2 = 500$, $h_2 = 2500000$, $h_1 = 10000$, $c_1 = 0.0309$, $c_2 = 0.00022$, $r_H = 0.4$, $d_{L_1} = 0.33$, $d_{L_2} = 0.26$, $d_H = 0.0012$, $d_A = 0.19$, and $T_d = 59.98$ (July- 100).

Figure S8(h) $\lambda_1 = 20000$, $\lambda_2 = 500$, $h_2 = 2500000$, $h_1 = 10000$, $c_1 = 0.0309$, $c_2 = 0.00021$, $r_H = 0.4$, $d_{L_1} = 0.33$, $d_{L_2} = 0.06$, $d_H = 0.0012$, $d_A = 0.14$, and $T_d = 59.98$ (August- 160).

Figure S8(i) $\lambda_1 = 20000$, $\lambda_2 = 500$, $h_2 = 2500000$, $h_1 = 10000$, $c_1 = 0.0309$, $c_2 = 0.000169$, $r_H = 0.4$, $d_{L_1} = 0.33$, $d_{L_2} = 0.06$, $d_H = 0.0012$, $d_A = 0.119$, and $T_d = 59.98$ (September- 135).

Figure S8(j) $\lambda_1 = 20000$, $\lambda_2 = 500$, $h_2 = 2500000$, $h_1 = 10000$, $c_1 = 0.0309$, $c_2 = 0.000183$, $r_H = 0.4$, $d_{L_1} = 0.33$, $d_{L_2} = 0.26$, $d_H = 0.0012$, $d_A = 0.19$, and $T_d = 59.98$ (October- 85).

Figure S8(k) $\lambda_1 = 20000$, $\lambda_2 = 500$, $h_2 = 2500000$, $h_1 = 10000$, $c_1 = 0.0309$, $c_2 = 0.00031$, $r_H = 0.4$, $d_{L_1} = 0.33$, $d_{L_2} = 0.26$, $d_H = 0.0012$, $d_A = 0.1129$, and $T_d = 59.98$ (November- 165).

Figure S8(l) $\lambda_1 = 20000$, $\lambda_2 = 500$, $h_2 = 2500000$, $h_1 = 10000$, $c_1 = 0.0309$, $c_2 = 0.00021$, $r_H = 0.4$, $d_{L_1} = 0.33$, $d_{L_2} = 0.26$, $d_H = 0.0012$, $d_A = 0.19$, and $T_d = 59.98$ (December- 95).

Figure S9(a) $\lambda_1 = 20000$, $\lambda_2 = 500$, $h_2 = 2500000$, $h_1 = 10000$, $c_1 = 0.0309$, $c_2 = 0.000098$, $r_H = 0.6$, $d_{L_1} = 0.33$, $d_{L_2} = 0.06$, $d_H = 0.0012$, $d_A = 0.119$, and $T_d = 59.98$ (January- 55).

Figure S9(b) $\lambda_1 = 20000$, $\lambda_2 = 500$, $h_2 = 2500000$, $h_1 = 10000$, $c_1 = 0.0309$, $c_2 = 0.000098$, $r_H = 0.7$, $d_{L_1} = 0.33$, $d_{L_2} = 0.06$, $d_H = 0.0012$, $d_A = 0.146$, and $T_d = 59.98$ (February- 45).

Figure S9(c) $\lambda_1 = 20000$, $\lambda_2 = 500$, $h_2 = 2500000$, $h_1 = 10000$, $c_1 = 0.0309$, $c_2 = 0.000098$, $r_H = 0.6$, $d_{L_1} = 0.33$,

$d_{L_2} = 0.3$, $d_H = 0.0012$, $d_A = 0.119$, and $T_d = 59.98$ (March- 40).

Figure S9(d) $\lambda_1 = 20000$, $\lambda_2 = 500$, $h_2 = 2500000$, $h_1 = 10000$, $c_1 = 0.0309$, $c_2 = 0.000098$, $r_H = 0.55$, $d_{L_1} = 0.33$, $d_{L_2} = 0.46$, $d_H = 0.0012$, $d_A = 0.19$, and $T_d = 59.98$ (April- 30).

Figure S9(e) $\lambda_1 = 20000$, $\lambda_2 = 500$, $h_2 = 2500000$, $h_1 = 10000$, $c_1 = 0.0309$, $c_2 = 0.0000998$, $r_H = 0.55$, $d_{L_1} = 0.33$, $d_{L_2} = 0.26$, $d_H = 0.0012$, $d_A = 0.119$, and $T_d = 59.98$ (May- 45).

Figure S9(f) $\lambda_1 = 20000$, $\lambda_2 = 500$, $h_2 = 2500000$, $h_1 = 10000$, $c_1 = 0.0309$, $c_2 = 0.000131$, $r_H = 0.4$, $d_{L_1} = 0.33$, $d_{L_2} = 0.06$, $d_H = 0.0012$, $d_A = 0.119$, and $T_d = 59.98$ (June- 105).

Figure S9(g) $\lambda_1 = 20000$, $\lambda_2 = 500$, $h_2 = 2500000$, $h_1 = 10000$, $c_1 = 0.0309$, $c_2 = 0.000246$, $r_H = 0.4$, $d_{L_1} = 0.33$, $d_{L_2} = 0.06$, $d_H = 0.0012$, $d_A = 0.119$, and $T_d = 59.98$ (July- 195).

Figure S9(h) $\lambda_1 = 20000$, $\lambda_2 = 500$, $h_2 = 2500000$, $h_1 = 10000$, $c_1 = 0.0309$, $c_2 = 0.000136$, $r_H = 0.4$, $d_{L_1} = 0.33$, $d_{L_2} = 0.06$, $d_H = 0.0012$, $d_A = 0.119$, and $T_d = 59.98$ (August- 110).

Figure S9(i) $\lambda_1 = 20000$, $\lambda_2 = 500$, $h_2 = 2500000$, $h_1 = 10000$, $c_1 = 0.0309$, $c_2 = 0.0001$, $r_H = 0.56$, $d_{L_1} = 0.33$, $d_{L_2} = 0.06$, $d_H = 0.0012$, $d_A = 0.119$, and $T_d = 59.98$ (September- 60).

Figure S9(j) $\lambda_1 = 20000$, $\lambda_2 = 500$, $h_2 = 2500000$, $h_1 = 10000$, $c_1 = 0.0309$, $c_2 = 0.000143$, $r_H = 0.4$, $d_{L_1} = 0.33$, $d_{L_2} = 0.06$, $d_H = 0.0012$, $d_A = 0.119$, and $T_d = 59.98$ (October- 115).

Figure S9(k) $\lambda_1 = 20000$, $\lambda_2 = 500$, $h_2 = 2500000$, $h_1 = 10000$, $c_1 = 0.0309$, $c_2 = 0.000163$, $r_H = 0.4$, $d_{L_1} = 0.33$, $d_{L_2} = 0.06$, $d_H = 0.0012$, $d_A = 0.119$, and $T_d = 59.98$ (November- 130).

Figure S9(l) $\lambda_1 = 20000$, $\lambda_2 = 500$, $h_2 = 2500000$, $h_1 = 10000$, $c_1 = 0.0309$, $c_2 = 0.000213$, $r_H = 0.4$, $d_{L_1} = 0.33$, $d_{L_2} = 0.06$, $d_H = 0.0012$, $d_A = 0.119$, and $T_d = 59.98$ (December- 170).

Figure S10(a) $\lambda_1 = 20000$, $\lambda_2 = 500$, $h_2 = 2500000$, $h_1 = 10000$, $c_1 = 0.0309$, $c_2 = 0.000118$, $r_H = 0.4$, $d_{L_1} = 0.33$, $d_{L_2} = 0.06$, $d_H = 0.0012$, $d_A = 0.119$, and $T_d = 59.98$ (January- 95).

Figure S10(b) $\lambda_1 = 20000$, $\lambda_2 = 500$, $h_2 = 2500000$, $h_1 = 10000$, $c_1 = 0.0309$, $c_2 = 0.000113$, $r_H = 0.46$, $d_{L_1} = 0.33$, $d_{L_2} = 0.06$, $d_H = 0.0012$, $d_A = 0.119$, and $T_d = 59.98$ (February- 80).

Figure S10(c) $\lambda_1 = 20000$, $\lambda_2 = 500$, $h_2 = 2500000$, $h_1 = 10000$, $c_1 = 0.0309$, $c_2 = 0.000112$, $r_H = 0.4$, $d_{L_1} = 0.33$, $d_{L_2} = 0.06$, $d_H = 0.0012$, $d_A = 0.119$, and $T_d = 59.98$ (March- 90).

Figure S10(d) $\lambda_1 = 20000$, $\lambda_2 = 500$, $h_2 = 2500000$, $h_1 = 10000$, $c_1 = 0.0309$, $c_2 = 0.000118$, $r_H = 0.4$, $d_{L_1} = 0.33$, $d_{L_2} = 0.06$, $d_H = 0.0012$, $d_A = 0.119$, and $T_d = 59.98$ (April- 95).

Figure S10(e) $\lambda_1 = 20000$, $\lambda_2 = 500$, $h_2 = 2500000$, $h_1 = 10000$, $c_1 = 0.0309$, $c_2 = 0.000143$, $r_H = 0.4$, $d_{L_1} = 0.33$, $d_{L_2} = 0.06$, $d_H = 0.0012$, $d_A = 0.119$, and $T_d = 59.98$ (May- 115).

Figure S10(f) $\lambda_1 = 20000$, $\lambda_2 = 500$, $h_2 = 2500000$, $h_1 = 10000$, $c_1 = 0.0309$, $c_2 = 0.000303$, $r_H = 0.4$, $d_{L_1} = 0.33$, $d_{L_2} = 0.06$, $d_H = 0.0012$, $d_A = 0.119$, and $T_d = 59.98$ (June- 240).

Figure S10(g) $\lambda_1 = 20000$, $\lambda_2 = 500$, $h_2 = 2500000$, $h_1 = 10000$, $c_1 = 0.0309$, $c_2 = 0.000316$, $r_H = 0.4$, $d_{L_1} = 0.33$, $d_{L_2} = 0.06$, $d_H = 0.0012$, $d_A = 0.119$, and $T_d = 59.98$ (July- 250).

Figure S10(h) $\lambda_1 = 20000$, $\lambda_2 = 500$, $h_2 = 2500000$, $h_1 = 10000$, $c_1 = 0.0309$, $c_2 = 0.00023$, $r_H = 0.4$, $d_{L_1} = 0.33$, $d_{L_2} = 0.06$, $d_H = 0.0012$, $d_A = 0.119$, and $T_d = 59.98$ (August- 185).

Figure S10(i) $\lambda_1 = 20000$, $\lambda_2 = 500$, $h_2 = 2500000$, $h_1 = 10000$, $c_1 = 0.0309$, $c_2 = 0.000245$, $r_H = 0.4$, $d_{L_1} = 0.33$, $d_{L_2} = 0.06$, $d_H = 0.0012$, $d_A = 0.119$, and $T_d = 59.98$ (September- 195).

Figure S10(j) $\lambda_1 = 20000$, $\lambda_2 = 500$, $h_2 = 2500000$, $h_1 = 10000$, $c_1 = 0.0309$, $c_2 = 0.000156$, $r_H = 0.4$, $d_{L_1} = 0.33$, $d_{L_2} = 0.06$, $d_H = 0.0012$, $d_A = 0.119$, and $T_d = 59.98$ (October- 125).

Figure S10(k) $\lambda_1 = 20000$, $\lambda_2 = 500$, $h_2 = 2500000$, $h_1 = 10000$, $c_1 = 0.0309$, $c_2 = 0.00013$, $r_H = 0.4$, $d_{L_1} = 0.33$, $d_{L_2} = 0.06$, $d_H = 0.0012$, $d_A = 0.119$, and $T_d = 59.98$ (November- 105).

Figure S10(l) $\lambda_1 = 20000$, $\lambda_2 = 500$, $h_2 = 2500000$, $h_1 = 10000$, $c_1 = 0.0309$, $c_2 = 0.000168$, $r_H = 0.4$, $d_{L_1} = 0.33$, $d_{L_2} = 0.06$, $d_H = 0.0012$, $d_A = 0.119$, and $T_d = 59.98$ (December- 135).

Figure S11(a) $\lambda_1 = 20000$, $\lambda_2 = 500$, $h_2 = 2500000$, $h_1 = 10000$, $c_1 = 0.0309$, $c_2 = 0.000125$, $r_H = 0.4$, $d_{L_1} = 0.33$, $d_{L_2} = 0.06$, $d_H = 0.0012$, $d_A = 0.119$, and $T_d = 59.98$ (January- 100).

Figure S11(b) $\lambda_1 = 20000$, $\lambda_2 = 500$, $h_2 = 2500000$, $h_1 = 10000$, $c_1 = 0.0309$, $c_2 = 0.000131$, $r_H = 0.4$, $d_{L_1} = 0.33$, $d_{L_2} = 0.06$, $d_H = 0.0012$, $d_A = 0.119$, and $T_d = 59.98$ (February- 105).

Figure S11(c) $\lambda_1 = 20000$, $\lambda_2 = 500$, $h_2 = 2500000$, $h_1 = 10000$, $c_1 = 0.0309$, $c_2 = 0.000125$, $r_H = 0.52$, $d_{L_1} = 0.33$, $d_{L_2} = 0.06$, $d_H = 0.0012$, $d_A = 0.119$, and $T_d = 59.98$ (March- 80).

Figure S11(d) $\lambda_1 = 20000$, $\lambda_2 = 500$, $h_2 = 2500000$, $h_1 = 10000$, $c_1 = 0.0309$, $c_2 = 0.000143$, $r_H = 0.4$, $d_{L_1} = 0.33$, $d_{L_2} = 0.06$, $d_H = 0.0012$, $d_A = 0.119$, and $T_d = 59.98$ (April- 115).

Figure S11(e) $\lambda_1 = 20000$, $\lambda_2 = 500$, $h_2 = 2500000$, $h_1 = 10000$, $c_1 = 0.0309$, $c_2 = 0.000181$, $r_H = 0.4$, $d_{L_1} = 0.33$, $d_{L_2} = 0.06$, $d_H = 0.0012$, $d_A = 0.119$, and $T_d = 59.98$ (May- 145).

Figure S11(f) $\lambda_1 = 20000$, $\lambda_2 = 500$, $h_2 = 2500000$, $h_1 = 10000$, $c_1 = 0.0309$, $c_2 = 0.000252$, $r_H = 0.4$, $d_{L_1} = 0.33$, $d_{L_2} = 0.06$, $d_H = 0.0012$, $d_A = 0.119$, and $T_d = 59.98$ (June- 200).

Figure S11(g) $\lambda_1 = 20000$, $\lambda_2 = 500$, $h_2 = 2500000$, $h_1 = 10000$, $c_1 = 0.0309$, $c_2 = 0.00017$, $r_H = 0.4$, $d_{L_1} = 0.33$, $d_{L_2} = 0.06$, $d_H = 0.0012$, $d_A = 0.119$, and $T_d = 59.98$ (July- 135).

Figure S11(h) $\lambda_1 = 20000$, $\lambda_2 = 500$, $h_2 = 2500000$, $h_1 = 10000$, $c_1 = 0.0309$, $c_2 = 0.000097$, $r_H = 0.697$, $d_{L_1} = 0.33$, $d_{L_2} = 0.09$, $d_H = 0.0012$, $d_A = 0.119$, and $T_d = 59.98$ (August- 45).

Figure S11(i) $\lambda_1 = 20000$, $\lambda_2 = 500$, $h_2 = 2500000$, $h_1 = 10000$, $c_1 = 0.0309$, $c_2 = 0.0001$, $r_H = 0.408$, $d_{L_1} = 0.33$, $d_{L_2} = 0.06$, $d_H = 0.0012$, $d_A = 0.119$, and $T_d = 59.98$ (September- 80).

Figure S11(j) $\lambda_1 = 20000$, $\lambda_2 = 500$, $h_2 = 2500000$, $h_1 = 10000$, $c_1 = 0.0309$, $c_2 = 0.000175$, $r_H = 0.4$, $d_{L_1} = 0.33$, $d_{L_2} = 0.06$, $d_H = 0.0012$, $d_A = 0.119$, and $T_d = 59.98$ (October- 140).

Figure S11(k) $\lambda_1 = 20000$, $\lambda_2 = 500$, $h_2 = 2500000$, $h_1 = 10000$, $c_1 = 0.0309$, $c_2 = 0.000195$, $r_H = 0.4$, $d_{L_1} = 0.33$, $d_{L_2} = 0.06$, $d_H = 0.0012$, $d_A = 0.119$, and $T_d = 59.98$ (November- 155).

Figure S11(l) $\lambda_1 = 20000$, $\lambda_2 = 500$, $h_2 = 2500000$, $h_1 = 10000$, $c_1 = 0.0309$, $c_2 = 0.000136$, $r_H = 0.4$, $d_{L_1} = 0.33$, $d_{L_2} = 0.06$, $d_H = 0.0012$, $d_A = 0.119$, and $T_d = 59.98$ (December- 110).

Figure S12(a) $\lambda_1 = 20000$, $\lambda_2 = 500$, $h_2 = 2500000$, $h_1 = 10000$, $c_1 = 0.0309$, $c_2 = 0.0001$, $r_H = 0.47$, $d_{L_1} = 0.33$, $d_{L_2} = 0.06$, $d_H = 0.0012$, $d_A = 0.119$, and $T_d = 59.98$ (January- 70).

Figure S12(b) $\lambda_1 = 20000$, $\lambda_2 = 500$, $h_2 = 2500000$, $h_1 = 10000$, $c_1 = 0.0309$, $c_2 = 0.0001$, $r_H = 0.56$, $d_{L_1} = 0.33$, $d_{L_2} = 0.06$, $d_H = 0.0012$, $d_A = 0.119$, and $T_d = 59.98$ (February- 60).

Figure S12(c) $\lambda_1 = 20000$, $\lambda_2 = 500$, $h_2 = 2500000$, $h_1 = 10000$, $c_1 = 0.0309$, $c_2 = 0.0001$, $r_H = 0.68$, $d_{L_1} = 0.33$, $d_{L_2} = 0.14$, $d_H = 0.0012$, $d_A = 0.119$, and $T_d = 59.98$ (March- 45).

Figure S12(d) $\lambda_1 = 20000$, $\lambda_2 = 500$, $h_2 = 2500000$, $h_1 = 10000$, $c_1 = 0.0309$, $c_2 = 0.0001$, $r_H = 0.56$, $d_{L_1} = 0.33$, $d_{L_2} = 0.06$, $d_H = 0.0012$, $d_A = 0.119$, and $T_d = 59.98$ (April- 60).

Figure S12(e) $\lambda_1 = 20000$, $\lambda_2 = 500$, $h_2 = 2500000$, $h_1 = 10000$, $c_1 = 0.0309$, $c_2 = 0.0001$, $r_H = 0.47$, $d_{L_1} = 0.33$, $d_{L_2} = 0.06$, $d_H = 0.0012$, $d_A = 0.119$, and $T_d = 59.98$ (May- 70).

Figure S12(f) $\lambda_1 = 20000$, $\lambda_2 = 500$, $h_2 = 2500000$, $h_1 = 10000$, $c_1 = 0.0309$, $c_2 = 0.0001$, $r_H = 0.68$, $d_{L_1} = 0.33$, $d_{L_2} = 0.06$, $d_H = 0.0012$, $d_A = 0.119$, and $T_d = 59.98$ (June- 50).

Figure S12(g) $\lambda_1 = 20000$, $\lambda_2 = 500$, $h_2 = 2500000$, $h_1 = 10000$, $c_1 = 0.0309$, $c_2 = 0.00014$, $r_H = 0.408$, $d_{L_1} = 0.33$, $d_{L_2} = 0.06$, $d_H = 0.0012$, $d_A = 0.119$, and $T_d = 59.98$ (July- 110).

Figure S12(h) $\lambda_1 = 20000$, $\lambda_2 = 500$, $h_2 = 2500000$, $h_1 = 10000$, $c_1 = 0.0309$, $c_2 = 0.0002$, $r_H = 0.412$, $d_{L_1} = 0.33$, $d_{L_2} = 0.06$, $d_H = 0.0012$, $d_A = 0.119$, and $T_d = 59.98$ (August- 155).

Figure S12(i) $\lambda_1 = 20000$, $\lambda_2 = 500$, $h_2 = 2500000$, $h_1 = 10000$, $c_1 = 0.0309$, $c_2 = 0.000184$, $r_H = 0.408$, $d_{L_1} = 0.33$, $d_{L_2} = 0.06$, $d_H = 0.0012$, $d_A = 0.119$, and $T_d = 59.98$ (September- 145).

Figure S12(j) $\lambda_1 = 20000$, $\lambda_2 = 500$, $h_2 = 2500000$, $h_1 = 10000$, $c_1 = 0.0309$, $c_2 = 0.000172$, $r_H = 0.408$, $d_{L_1} = 0.33$, $d_{L_2} = 0.06$, $d_H = 0.0012$, $d_A = 0.119$, and $T_d = 59.98$ (October- 135).

Figure S12(k) $\lambda_1 = 20000$, $\lambda_2 = 500$, $h_2 = 2500000$, $h_1 = 10000$, $c_1 = 0.0309$, $c_2 = 0.000243$, $r_H = 0.408$, $d_{L_1} = 0.33$, $d_{L_2} = 0.06$, $d_H = 0.0012$, $d_A = 0.119$, and $T_d = 59.98$ (November- 190).

Figure S12(l) $\lambda_1 = 20000$, $\lambda_2 = 500$, $h_2 = 2500000$, $h_1 = 10000$, $c_1 = 0.0309$, $c_2 = 0.00016$, $r_H = 0.408$, $d_{L_1} = 0.33$, $d_{L_2} = 0.06$, $d_H = 0.0012$, $d_A = 0.119$, and $T_d = 59.98$ (December- 125).

Figure S13(a) $\lambda_1 = 20000$, $\lambda_2 = 500$, $h_2 = 2500000$, $h_1 = 10000$, $c_1 = 0.0309$, $c_2 = 0.0001$, $r_H = 0.508$, $d_{L_1} = 0.33$, $d_{L_2} = 0.06$, $d_H = 0.0012$, $d_A = 0.119$, and $T_d = 59.98$ (January- 65).

Figure S13(b) $\lambda_1 = 20000$, $\lambda_2 = 500$, $h_2 = 2500000$, $h_1 = 10000$, $c_1 = 0.0309$, $c_2 = 0.00011$, $r_H = 0.68$, $d_{L_1} = 0.33$, $d_{L_2} = 0.06$, $d_H = 0.0012$, $d_A = 0.119$, and $T_d = 59.98$ (February- 55).

Figure S13(c) $\lambda_1 = 20000$, $\lambda_2 = 500$, $h_2 = 2500000$, $h_1 = 10000$, $c_1 = 0.0309$, $c_2 = 0.00009634$, $r_H = 0.717$, $d_{L_1} = 0.336$, $d_{L_2} = 0.48$, $d_H = 0.00138$, $d_A = 0.2099$, and $T_d = 59.92$ (March- 20).

Figure S13(d) $\lambda_1 = 20000$, $\lambda_2 = 500$, $h_2 = 2500000$, $h_1 = 10000$, $c_1 = 0.0309$, $c_2 = 0.0001$, $r_H = 0.7$, $d_{L_1} = 0.33$, $d_{L_2} = 0.36$, $d_H = 0.0012$, $d_A = 0.18$, and $T_d = 59.98$ (April- 30).

Figure S13(e) $\lambda_1 = 20000$, $\lambda_2 = 500$, $h_2 = 2500000$, $h_1 = 10000$, $c_1 = 0.0309$, $c_2 = 0.000124$, $r_H = 0.48$, $d_{L_1} = 0.33$, $d_{L_2} = 0.06$, $d_H = 0.0012$, $d_A = 0.119$, and $T_d = 59.98$ (May- 85).

Figure S13(f) $\lambda_1 = 20000$, $\lambda_2 = 500$, $h_2 = 2500000$, $h_1 = 10000$, $c_1 = 0.0309$, $c_2 = 0.000127$, $r_H = 0.408$, $d_{L_1} = 0.33$, $d_{L_2} = 0.06$, $d_H = 0.0012$, $d_A = 0.119$, and $T_d = 59.98$ (June- 100).

Figure S13(g) $\lambda_1 = 20000$, $\lambda_2 = 500$, $h_2 = 2500000$, $h_1 = 10000$, $c_1 = 0.0309$, $c_2 = 0.000152$, $r_H = 0.408$, $d_{L_1} = 0.33$, $d_{L_2} = 0.06$, $d_H = 0.0012$, $d_A = 0.119$, and $T_d = 59.98$ (July- 120).

Figure S13(h) $\lambda_1 = 20000$, $\lambda_2 = 500$, $h_2 = 2500000$, $h_1 = 10000$, $c_1 = 0.0309$, $c_2 = 0.000127$, $r_H = 0.408$, $d_{L_1} = 0.33$, $d_{L_2} = 0.06$, $d_H = 0.0012$, $d_A = 0.119$, and $T_d = 59.98$ (August- 100).

Figure S13(i) $\lambda_1 = 20000$, $\lambda_2 = 500$, $h_2 = 2500000$, $h_1 = 10000$, $c_1 = 0.0309$, $c_2 = 0.000165$, $r_H = 0.408$, $d_{L_1} = 0.33$, $d_{L_2} = 0.06$, $d_H = 0.0012$, $d_A = 0.119$, and $T_d = 59.98$ (September- 130).

Figure S13(j) $\lambda_1 = 20000$, $\lambda_2 = 500$, $h_2 = 2500000$, $h_1 = 10000$, $c_1 = 0.0309$, $c_2 = 0.000178$, $r_H = 0.408$, $d_{L_1} = 0.33$,

$d_{L_2} = 0.06$, $d_H = 0.0012$, $d_A = 0.119$, and $T_d = 59.98$ (October- 140).

Figure S13(k) $\lambda_1 = 20000$, $\lambda_2 = 500$, $h_2 = 2500000$, $h_1 = 10000$, $c_1 = 0.0309$, $c_2 = 0.00014$, $r_H = 0.408$, $d_{L_1} = 0.33$, $d_{L_2} = 0.06$, $d_H = 0.0012$, $d_A = 0.119$, and $T_d = 59.98$ (November- 110).

Figure S13(l) $\lambda_1 = 20000$, $\lambda_2 = 500$, $h_2 = 2500000$, $h_1 = 10000$, $c_1 = 0.0309$, $c_2 = 0.000108$, $r_H = 0.408$, $d_{L_1} = 0.33$, $d_{L_2} = 0.06$, $d_H = 0.0012$, $d_A = 0.119$, and $T_d = 59.98$ (December- 85).

Figure S14(a) $\lambda_1 = 20000$, $\lambda_2 = 500$, $h_2 = 2500000$, $h_1 = 10000$, $c_1 = 0.0309$, $c_2 = 0.0001$, $r_H = 0.432$, $d_{L_1} = 0.33$, $d_{L_2} = 0.06$, $d_H = 0.0012$, $d_A = 0.119$, and $T_d = 59.98$ (January- 75).

Figure S14(b) $\lambda_1 = 20000$, $\lambda_2 = 500$, $h_2 = 2500000$, $h_1 = 10000$, $c_1 = 0.0309$, $c_2 = 0.000139$, $r_H = 0.408$, $d_{L_1} = 0.33$, $d_{L_2} = 0.06$, $d_H = 0.0012$, $d_A = 0.119$, and $T_d = 59.98$ (Feb - 110).

Figure S14(c) $\lambda_1 = 20000$, $\lambda_2 = 500$, $h_2 = 2500000$, $h_1 = 10000$, $c_1 = 0.0309$, $c_2 = 0.0001$, $r_H = 0.558$, $d_{L_1} = 0.33$, $d_{L_2} = 0.06$, $d_H = 0.0012$, $d_A = 0.119$, and $T_d = 59.98$ (March- 60).

Figure S14(d) $\lambda_1 = 20000$, $\lambda_2 = 500$, $h_2 = 2500000$, $h_1 = 10000$, $c_1 = 0.0309$, $c_2 = 0.0001$, $r_H = 0.63$, $d_{L_1} = 0.33$, $d_{L_2} = 0.29$, $d_H = 0.0012$, $d_A = 0.119$, and $T_d = 59.98$ (April- 40).

Figure S14(e) $\lambda_1 = 20000$, $\lambda_2 = 500$, $h_2 = 2500000$, $h_1 = 10000$, $c_1 = 0.0309$, $c_2 = 0.000098$, $r_H = 0.68$, $d_{L_1} = 0.33$, $d_{L_2} = 0.22$, $d_H = 0.0012$, $d_A = 0.119$, and $T_d = 59.98$ (May- 40).

Figure S14(f) $\lambda_1 = 20000$, $\lambda_2 = 500$, $h_2 = 2500000$, $h_1 = 10000$, $c_1 = 0.0309$, $c_2 = 0.000165$, $r_H = 0.408$, $d_{L_1} = 0.33$, $d_{L_2} = 0.06$, $d_H = 0.0012$, $d_A = 0.119$, and $T_d = 59.98$ (June- 130).

Figure S14(g) $\lambda_1 = 20000$, $\lambda_2 = 500$, $h_2 = 2500000$, $h_1 = 10000$, $c_1 = 0.0309$, $c_2 = 0.00019$, $r_H = 0.408$, $d_{L_1} = 0.33$, $d_{L_2} = 0.06$, $d_H = 0.0012$, $d_A = 0.119$, and $T_d = 59.98$ (July- 150).

Figure S14(h) $\lambda_1 = 20000$, $\lambda_2 = 500$, $h_2 = 2500000$, $h_1 = 10000$, $c_1 = 0.0309$, $c_2 = 0.00023$, $r_H = 0.408$, $d_{L_1} = 0.33$, $d_{L_2} = 0.06$, $d_H = 0.0012$, $d_A = 0.119$, and $T_d = 59.98$ (August- 180).

Figure S14(i) $\lambda_1 = 20000$, $\lambda_2 = 500$, $h_2 = 2500000$, $h_1 = 10000$, $c_1 = 0.0309$, $c_2 = 0.00023$, $r_H = 0.408$, $d_{L_1} = 0.33$, $d_{L_2} = 0.06$, $d_H = 0.0012$, $d_A = 0.119$, and $T_d = 59.98$ (September- 180).

Figure S14(j) $\lambda_1 = 20000$, $\lambda_2 = 500$, $h_2 = 2500000$, $h_1 = 10000$, $c_1 = 0.0309$, $c_2 = 0.00027$, $r_H = 0.408$, $d_{L_1} = 0.33$, $d_{L_2} = 0.06$, $d_H = 0.0012$, $d_A = 0.119$, and $T_d = 59.98$ (October- 210).

Figure S14(k) $\lambda_1 = 20000$, $\lambda_2 = 500$, $h_2 = 2500000$, $h_1 = 10000$, $c_1 = 0.0309$, $c_2 = 0.000366$, $r_H = 0.408$, $d_{L_1} = 0.33$, $d_{L_2} = 0.06$, $d_H = 0.0012$, $d_A = 0.119$, and $T_d = 59.98$ (November- 285).

Figure S14(l) $\lambda_1 = 20000$, $\lambda_2 = 500$, $h_2 = 2500000$, $h_1 = 10000$, $c_1 = 0.0309$, $c_2 = 0.00027$, $r_H = 0.408$, $d_{L_1} = 0.33$, $d_{L_2} = 0.06$, $d_H = 0.0012$, $d_A = 0.119$, and $T_d = 59.98$ (December- 280).

S4 Figures

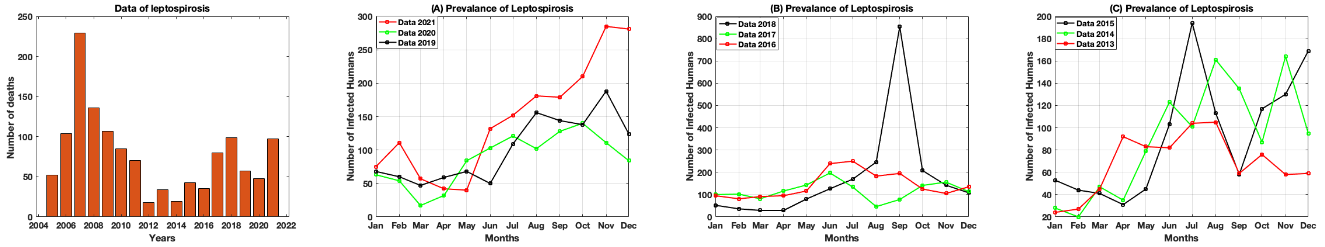

(a) (b) (c) (d)

Figure S1. *Figure (a) illustrates the temporal patterns of human deaths attributed to leptospirosis in the region from 2005 to 2021, while figures (b)-(d) depict the monthly trends in the number of leptospirosis cases in Kerala, India, from 2013 to 2021.*

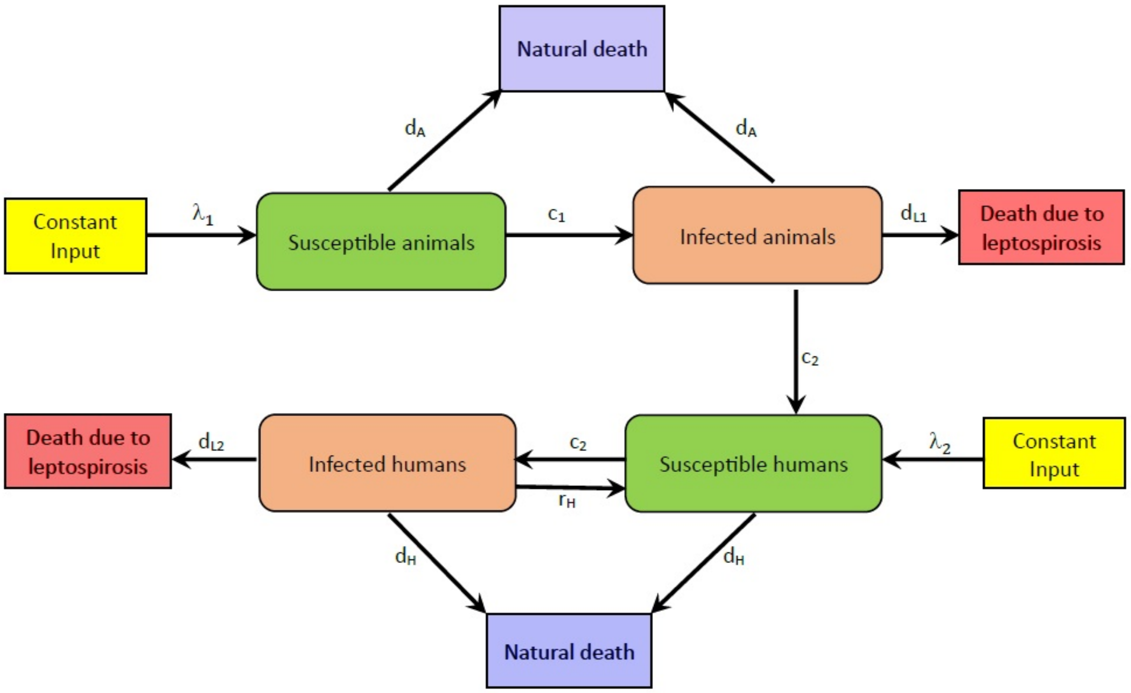

Figure S2. *Flowchart of the leptospirosis dynamics between human and animal populations. The black arrow shows the transmission route of leptospirosis from infected animal population* (I_A) *to susceptible humans* (S_H) *and susceptible animals* (S_A) *with a transmission rate of* (c_2) *to humans and* (c_1) *to animals. Infected humans that recover from the infections became susceptible again with a recovery rate* (r_H)*.*

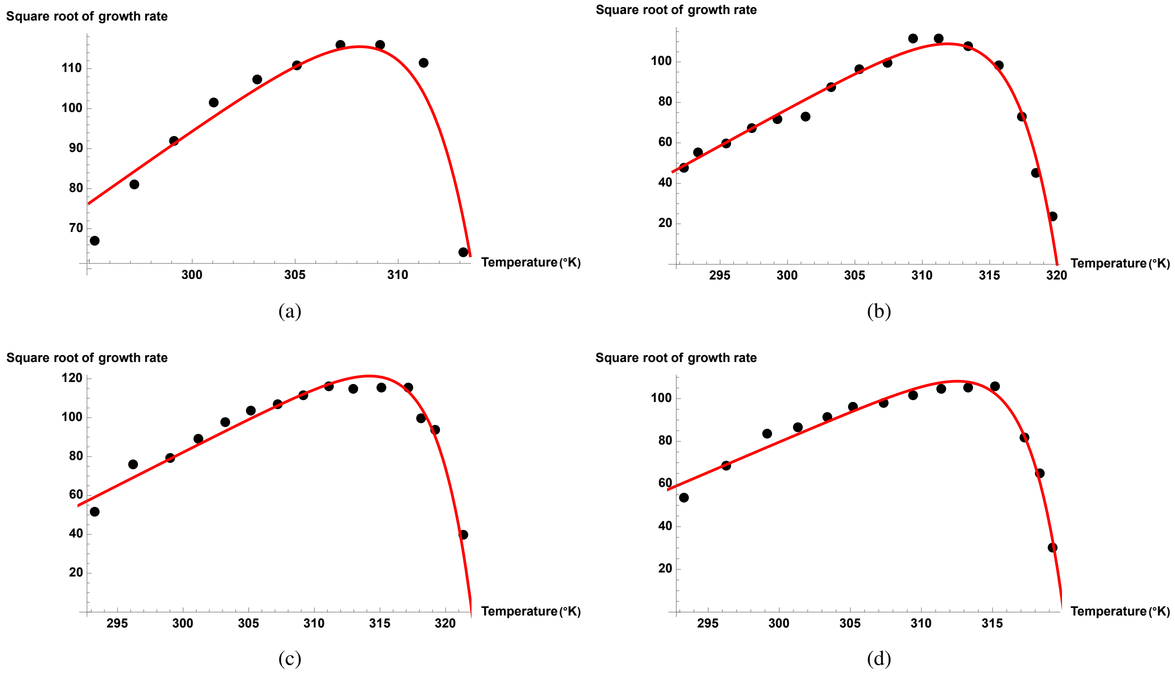

Figure S3. *The figures (a)-(d) shows the curve of best-fit for the square root of growth rate of Serratia marcescens, Pseudomonas fluorescens, Escherichia coli, and Pseudomonas aeruginosa respectively, to estimate the parameters α, β in Ratkowsky's model.*

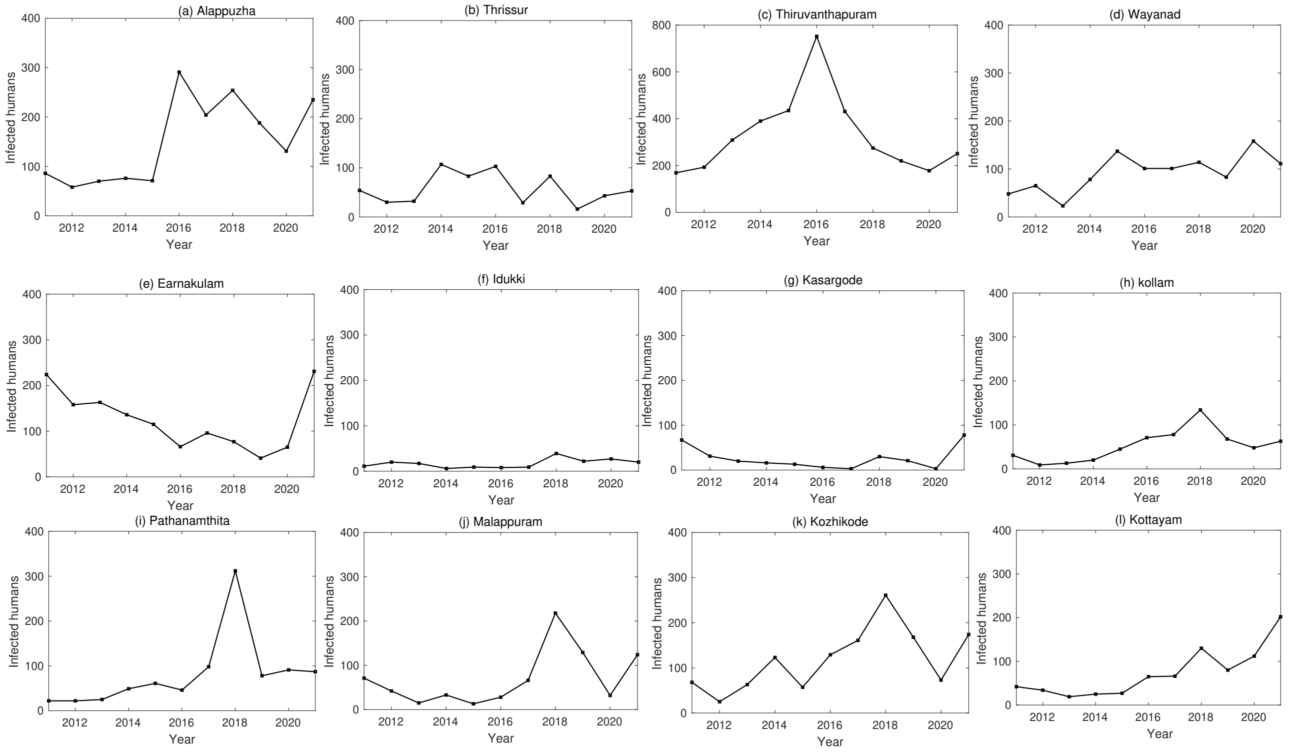

Figure S4. *The figures illustrate the temporal patterns of leptospirosis infections among humans in Kerala (district-wise) over 11 years [39].*

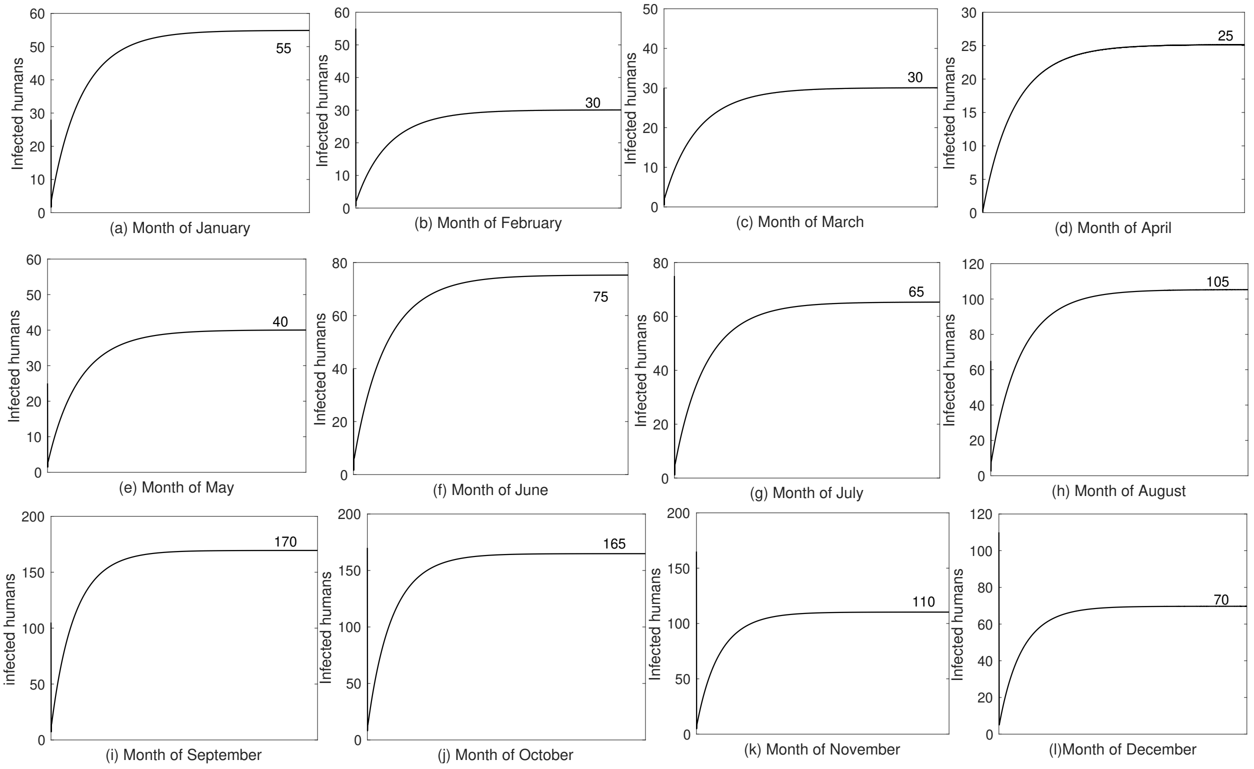

Figure S5. *The figures show the predicted monthly number of leptospirosis cases in Kerala, India, for the years 2011.*

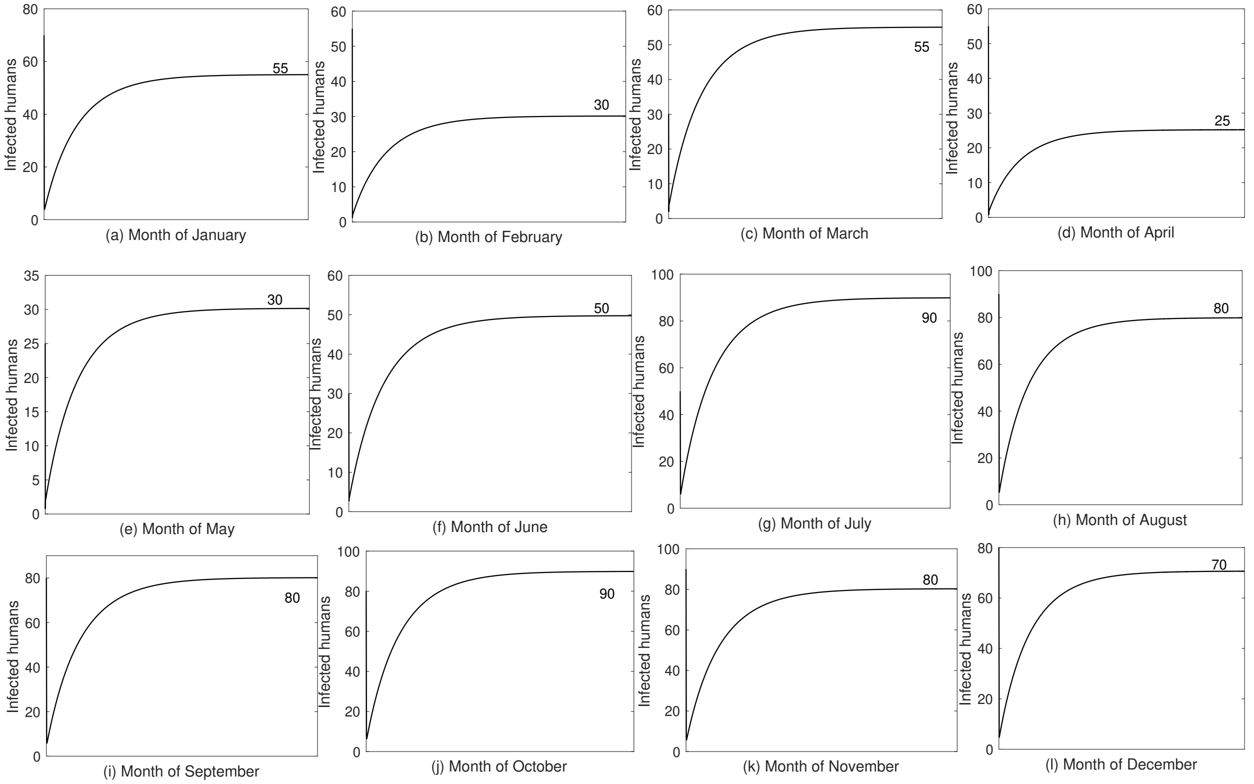

Figure S6. *The figures show the predicted monthly number of leptospirosis cases in Kerala, India, for the years 2012.*

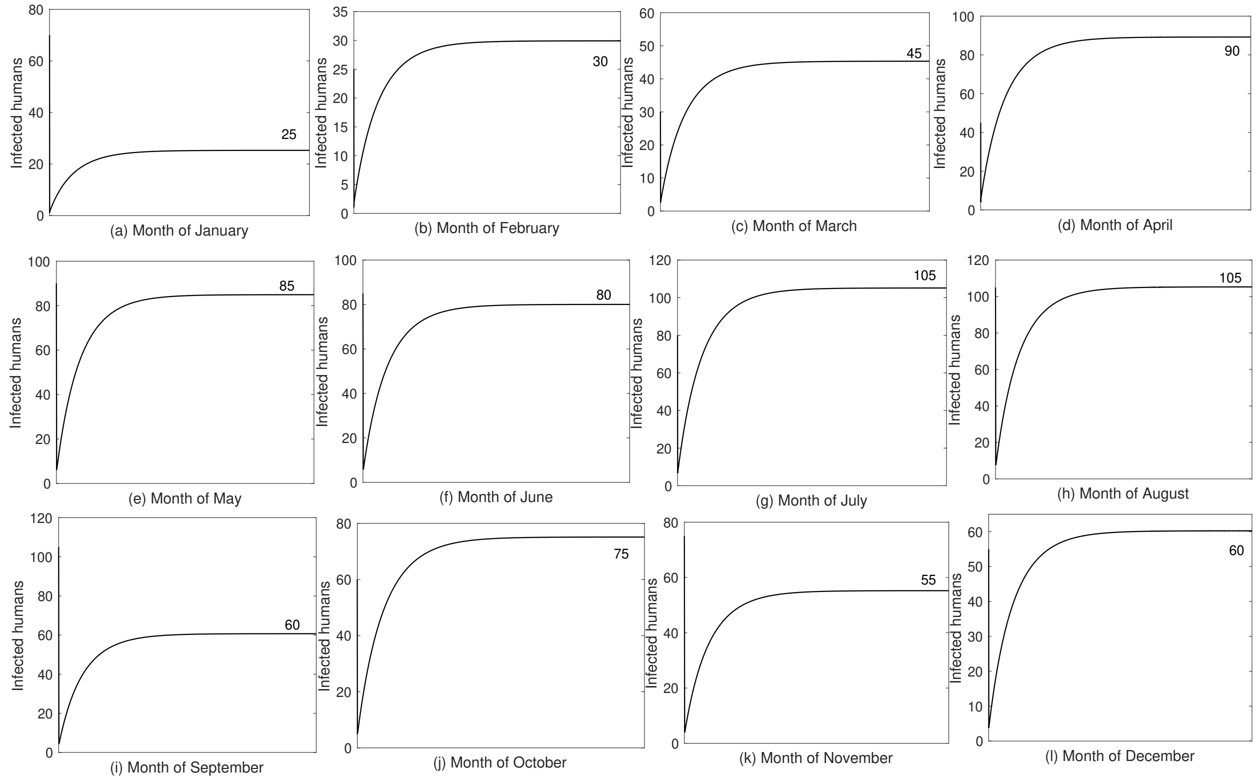

Figure S7. *The figures show the predicted monthly number of leptospirosis cases in Kerala, India, for the years 2013.*

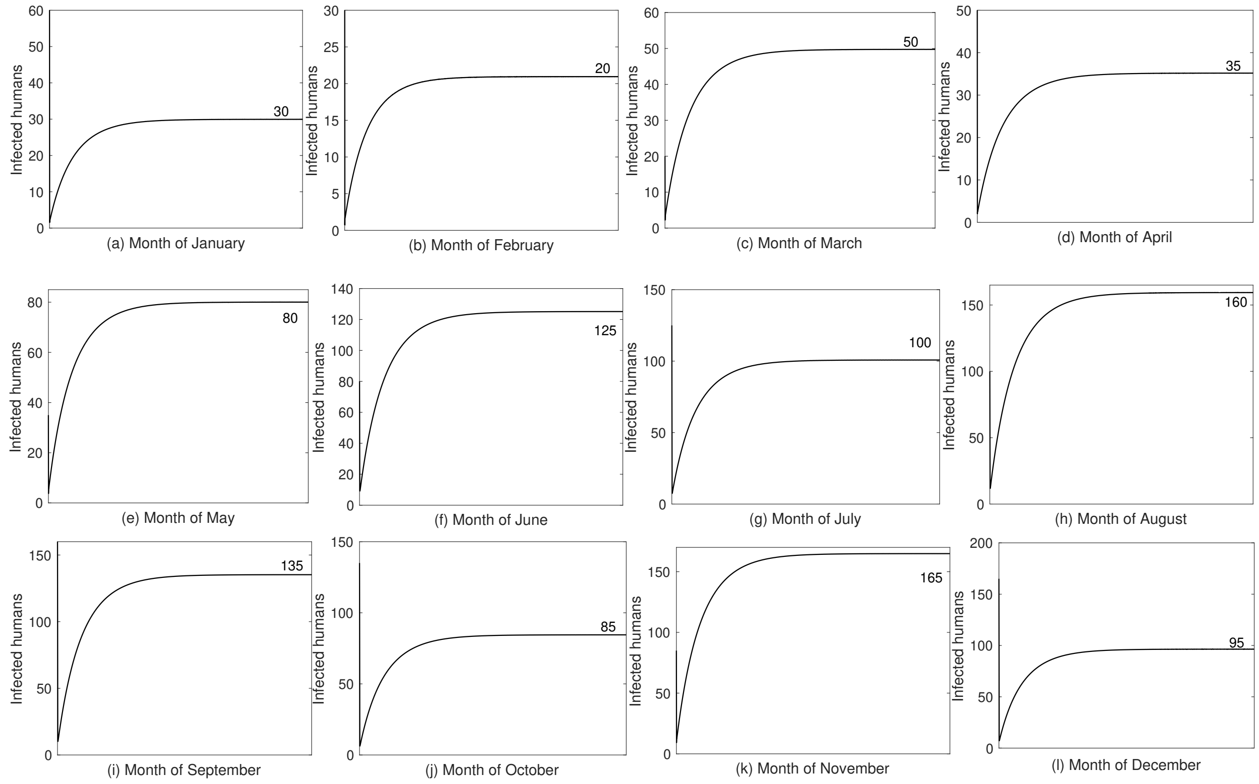

Figure S8. *The figures show the predicted monthly number of leptospirosis cases in Kerala, India, for the years 2014.*

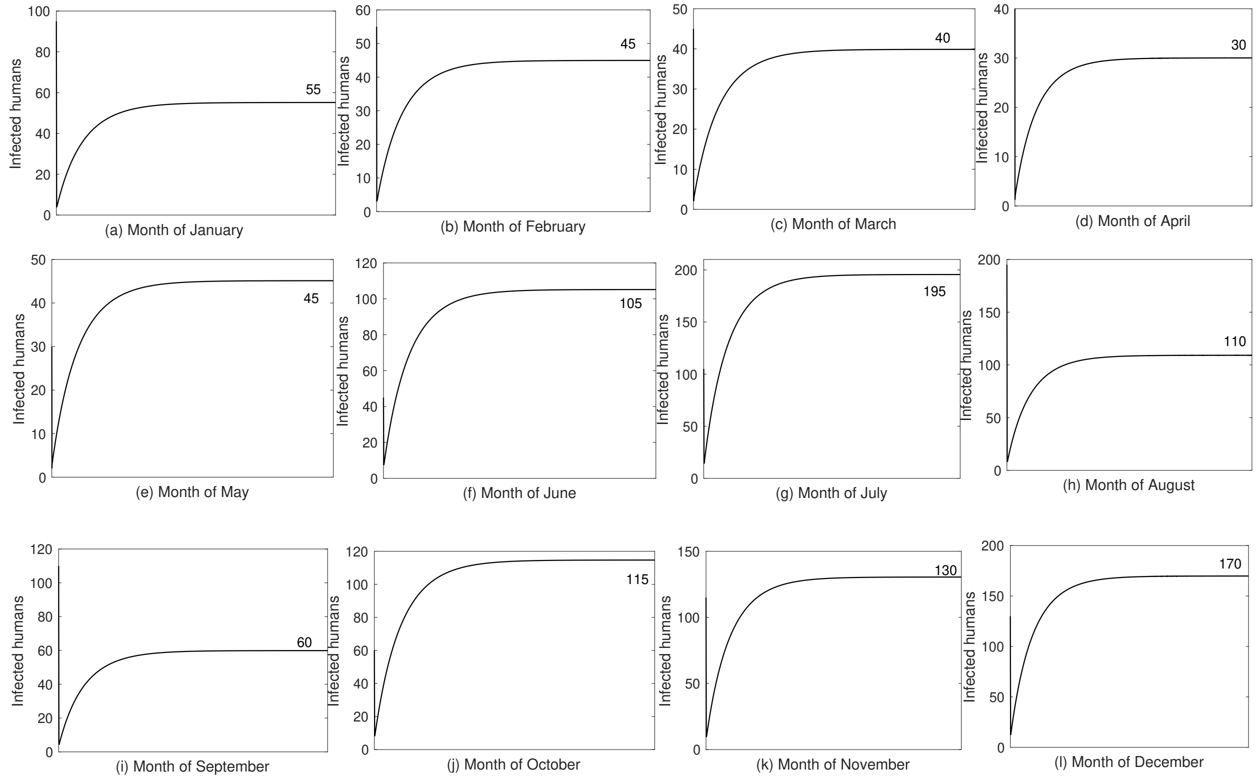

Figure S9. *The figures show the predicted monthly number of leptospirosis cases in Kerala, India, for the years 2015.*

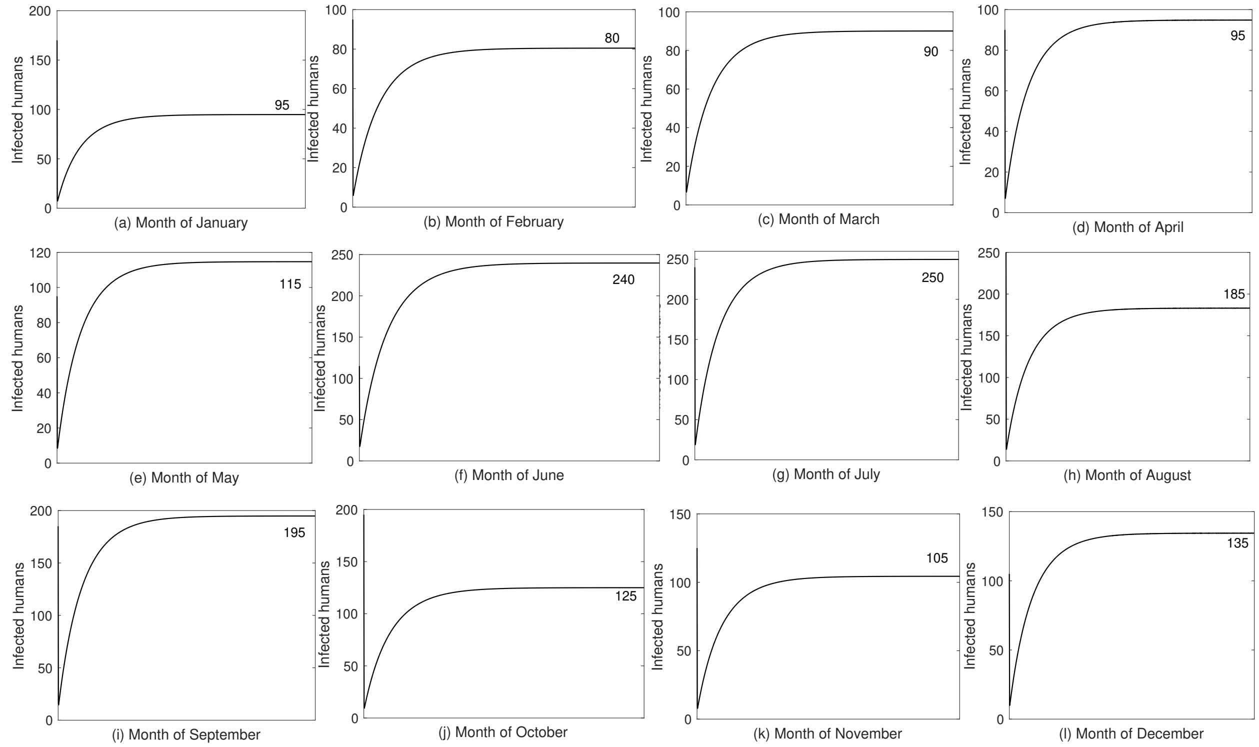

Figure S10. *The figures show the predicted monthly number of leptospirosis cases in Kerala, India, for the years 2016.*

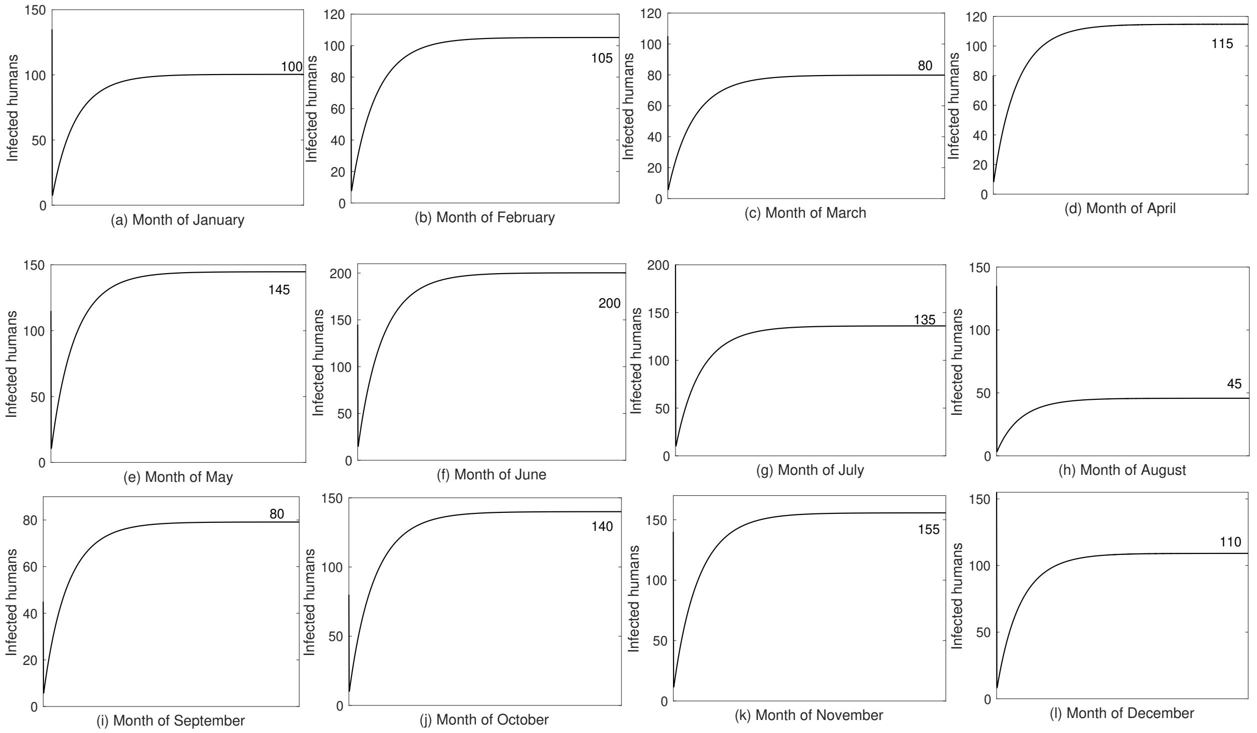

Figure S11. *The figure show the predicted monthly number of leptospirosis cases in Kerala, India, for the years 2017.*

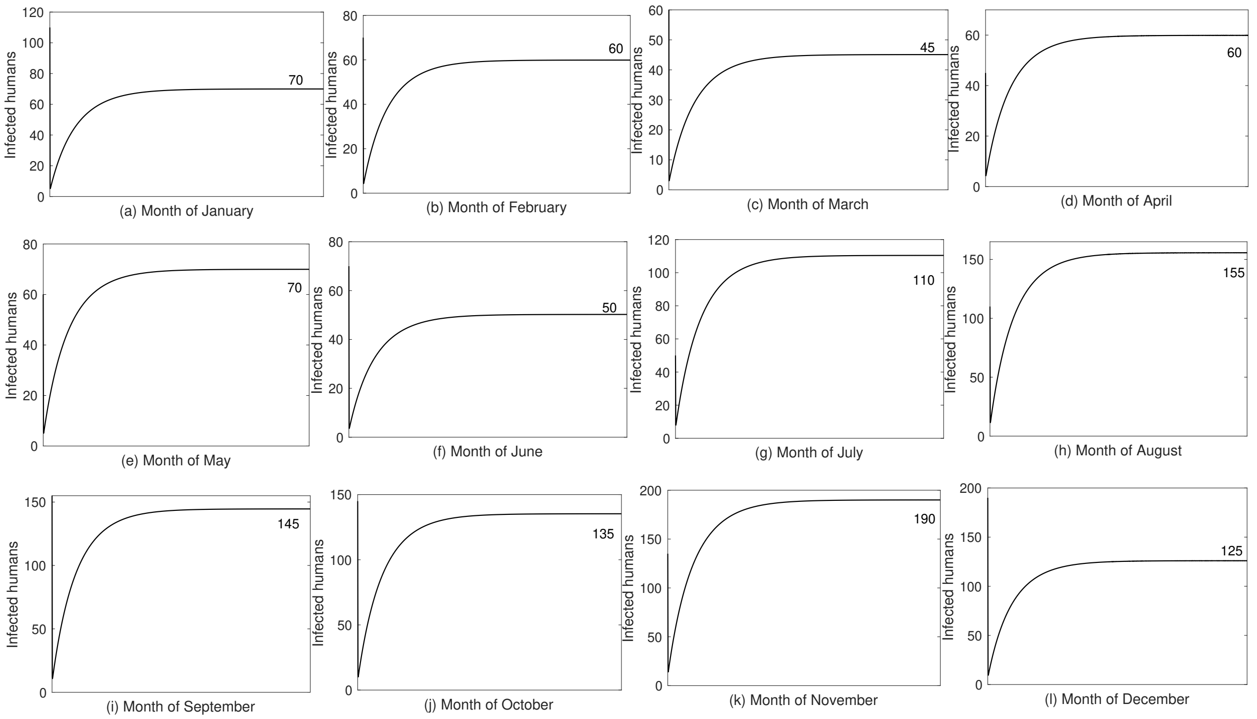

Figure S12. *The figures show the predicted monthly number of leptospirosis cases in Kerala, India, for the years 2019.*

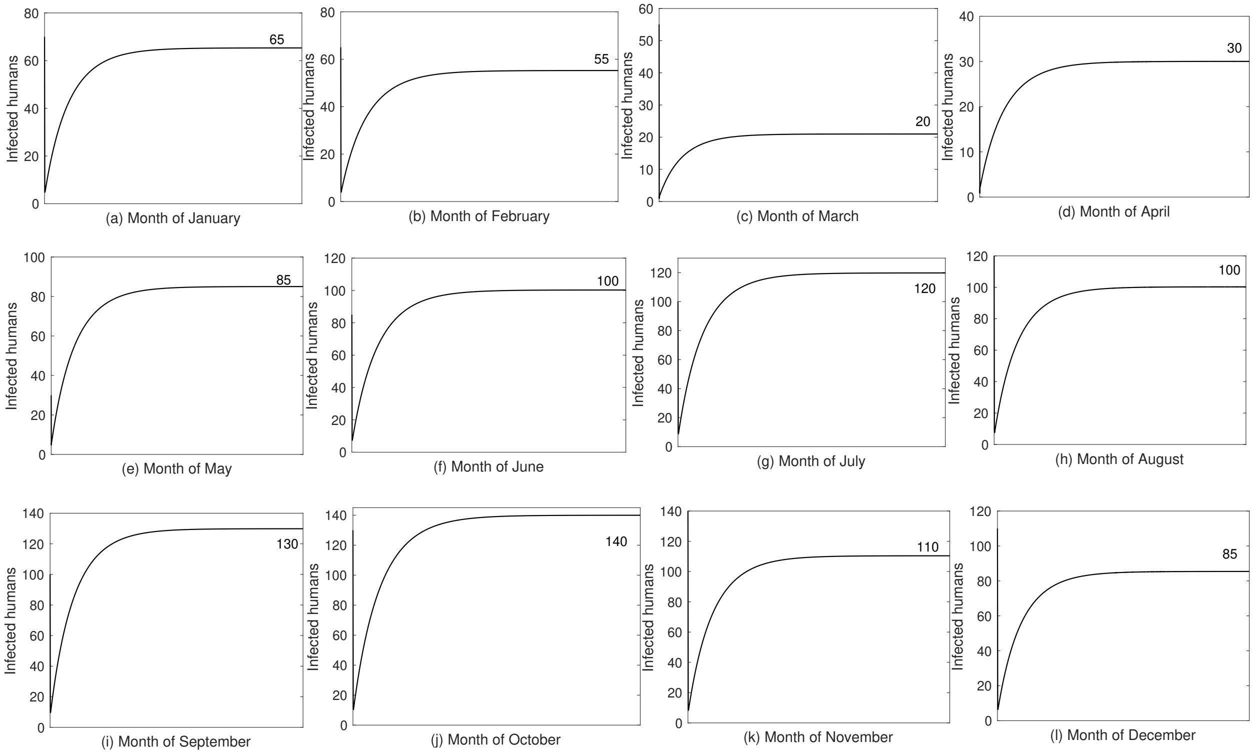

Figure S13. *The figures show the predicted monthly number of leptospirosis cases in Kerala, India, for the years 2020.*

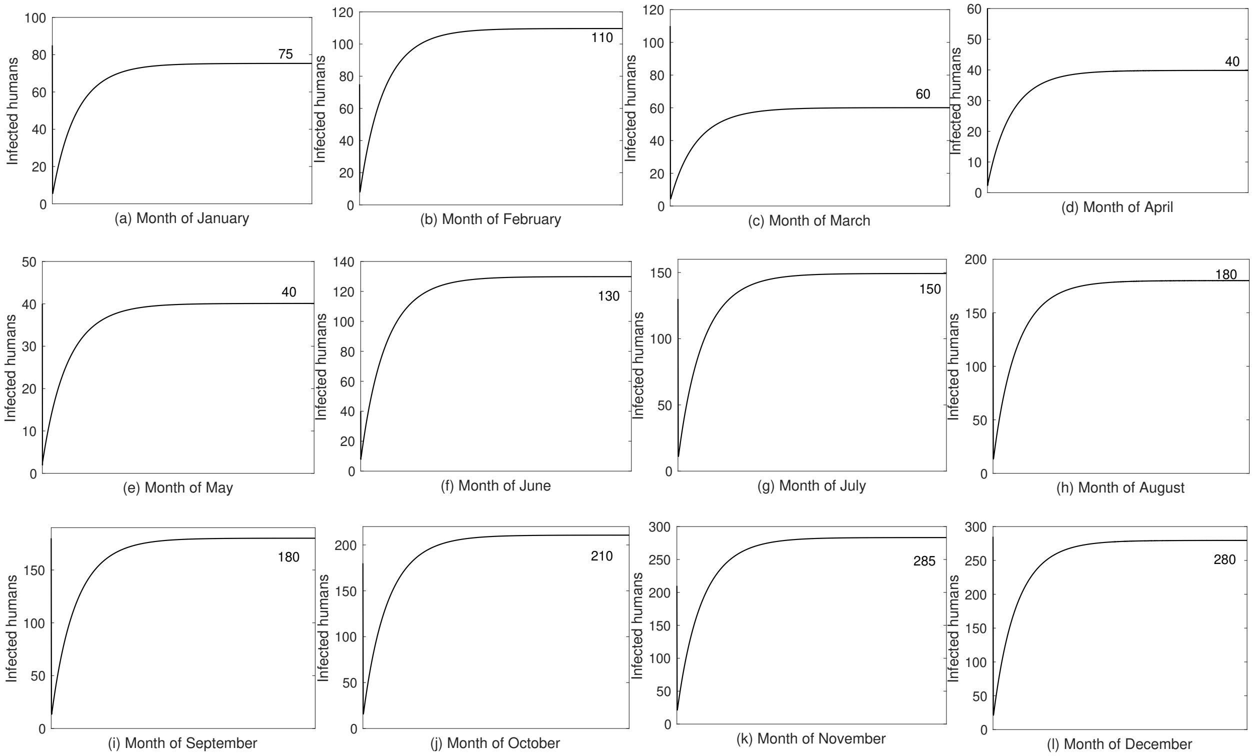

Figure S14. *The figures show the predicted monthly number of leptospirosis cases in Kerala, India, for the years 2021.*
